# Some like it hot: adaptation to the urban heat island in common dandelion

**DOI:** 10.1093/evlett/qrae040

**Published:** 2024-07-31

**Authors:** Yannick Woudstra, Ron Kraaiveld, Alger Jorritsma, Kitty Vijverberg, Slavica Ivanovic, Roy Erkens, Heidrun Huber, Barbara Gravendeel, Koen J F Verhoeven

**Affiliations:** Department of Terrestrial Ecology, Netherlands Institute of Ecology, Wageningen, The Netherlands; Naturalis Biodiversity Center, Evolutionary Ecology, Leiden, The Netherlands; Department of Terrestrial Ecology, Netherlands Institute of Ecology, Wageningen, The Netherlands; Radboud University Nijmegen, Radboud Institute of Biological and Environmental Sciences, Nijmegen, The Netherlands; Department of Terrestrial Ecology, Netherlands Institute of Ecology, Wageningen, The Netherlands; Laboratory of Genetics, Wageningen University & Research, Wageningen, The Netherlands; Naturalis Biodiversity Center, Evolutionary Ecology, Leiden, The Netherlands; Radboud University Nijmegen, Radboud Institute of Biological and Environmental Sciences, Nijmegen, The Netherlands; Department of Terrestrial Ecology, Netherlands Institute of Ecology, Wageningen, The Netherlands; Maastricht University, Maastricht Science Programme, Maastricht, The Netherlands; Maastricht University, System Earth Science, Maastricht, The Netherlands; Radboud University Nijmegen, Radboud Institute of Biological and Environmental Sciences, Nijmegen, The Netherlands; Naturalis Biodiversity Center, Evolutionary Ecology, Leiden, The Netherlands; Radboud University Nijmegen, Radboud Institute of Biological and Environmental Sciences, Nijmegen, The Netherlands; Department of Terrestrial Ecology, Netherlands Institute of Ecology, Wageningen, The Netherlands

**Keywords:** urbanization, climate change, anthropogenic environments, selection, Asteraceae, apomixis

## Abstract

The Urban Heat Island Effect (UHIE) is a globally consistent pressure on biological species living in cities. Adaptation to the UHIE may be necessary for urban wild flora to persist in cities, but experimental evidence is scarce. Here, we report evidence of adaptive evolution in a perennial plant species in response to the UHIE. We collected seeds from common dandelion (*Taraxacum officinale*) individuals along an urban–rural gradient in the city of Amsterdam (The Netherlands). In common-environment greenhouse experiments, we assessed the effect of elevated temperatures on plant growth and the effect of vernalization treatments on flowering phenology. We found that urban plants accumulate more biomass at higher temperatures and require shorter vernalization periods, corresponding to milder winters, to induce flowering compared to rural plants. Differentiation was also observed between different intra-urban subhabitats, with park plants displaying a higher vernalization requirement than street plants. Our results show genetic differentiation between urban and rural dandelions in temperature-dependent growth and phenology, consistent with adaptive divergence in response to the UHIE. Adaptation to the UHIE may be a potential explanation for the persistence of dandelions in urban environments.

The urban environment is a relatively novel ecosystem that has introduced a significant environmental change to the co-inhabiting and surrounding wild species (Johnson & Munshi-South, 2017). Considering the many different stakeholders in urban environments and the rapidity of global urbanization, it is important to understand the adaptive potential of nature to city life (Rivkin et al., 2019), especially for indigenous species. Eco-evolutionary experiments involving comparisons between rural and urban counterparts can demonstrate if, how, and at which rate natural species can survive and adapt to urban environments, important for the safeguarding of ecosystem services (Wong et al., 2021). The urban heat island effect (UHIE) is the most well-documented and consistent environmental pressure exerted by urbanization on nature in and around cities (Tzavali et al., 2015). Adaptation to urban heat can therefore be predicted as a hallmark of urban evolution. This has been demonstrated in fungi (McLean et al., 2005) and a variety of animal species (Brans et al., 2017; Campbell-Staton et al., 2020; Diamond et al., 2018; Kerstes et al., 2019; Piano et al., 2017; Tüzün et al., 2017), providing general evidence for the importance of adaptation to the UHIE. However, adaptation of plants to the UHIE is understudied (Lambert et al., 2021), despite vegetation being the most important factor in UHIE mitigation. Vegetation provides considerable cooling of urban surface and air temperatures through shading and covering of heat-retaining materials, evapotranspiration, and increased albedo (Tan et al., 2021).

Temperatures in cities are higher compared to rural areas, caused by the retention and re-radiation of heat from solar radiations by buildings and by the emission of heat from anthropogenic heat sources (Rizwan et al., 2008). The intensity of the UHIE is highly seasonal (Manoli et al., 2020) and mainly depends on the density of buildings, intensity of traffic, presence of industrial activity, and the mitigation of heat by water and vegetation (Tzavali et al., 2015). For example, in the capital of the Netherlands, Amsterdam, the UHIE leads to an annual average temperature increase of >2 °C in the city center (Figure 1A), while a hot summer day can elevate this difference to up to 9 °C for ambient temperatures and up to 20 °C for surface temperatures (Van Der Hoeven & Wandl, 2013). These conditions would selectively favor species (Piano et al., 2017) and genotypes that are optimized for growth at elevated temperatures. The UHIE can also affect the timing and duration of the growing season (Kabano et al., 2021), creating differential phenological optima between urban and rural habitats. In cities in the Northern Hemisphere, winter surface temperatures are on average 1–2 °C higher and warm up earlier in the year compared to rural sites (Manoli et al., 2020). An important determinant of flowering phenology is vernalization: extended exposure to low temperatures is required to enable the transition to flowering in many temperate plants (Samach & Coupland, 2000). Differential selection on vernalization requirements and flowering time can therefore be expected along urban–rural clines in relation to the UHIE.

**Figure 1.**
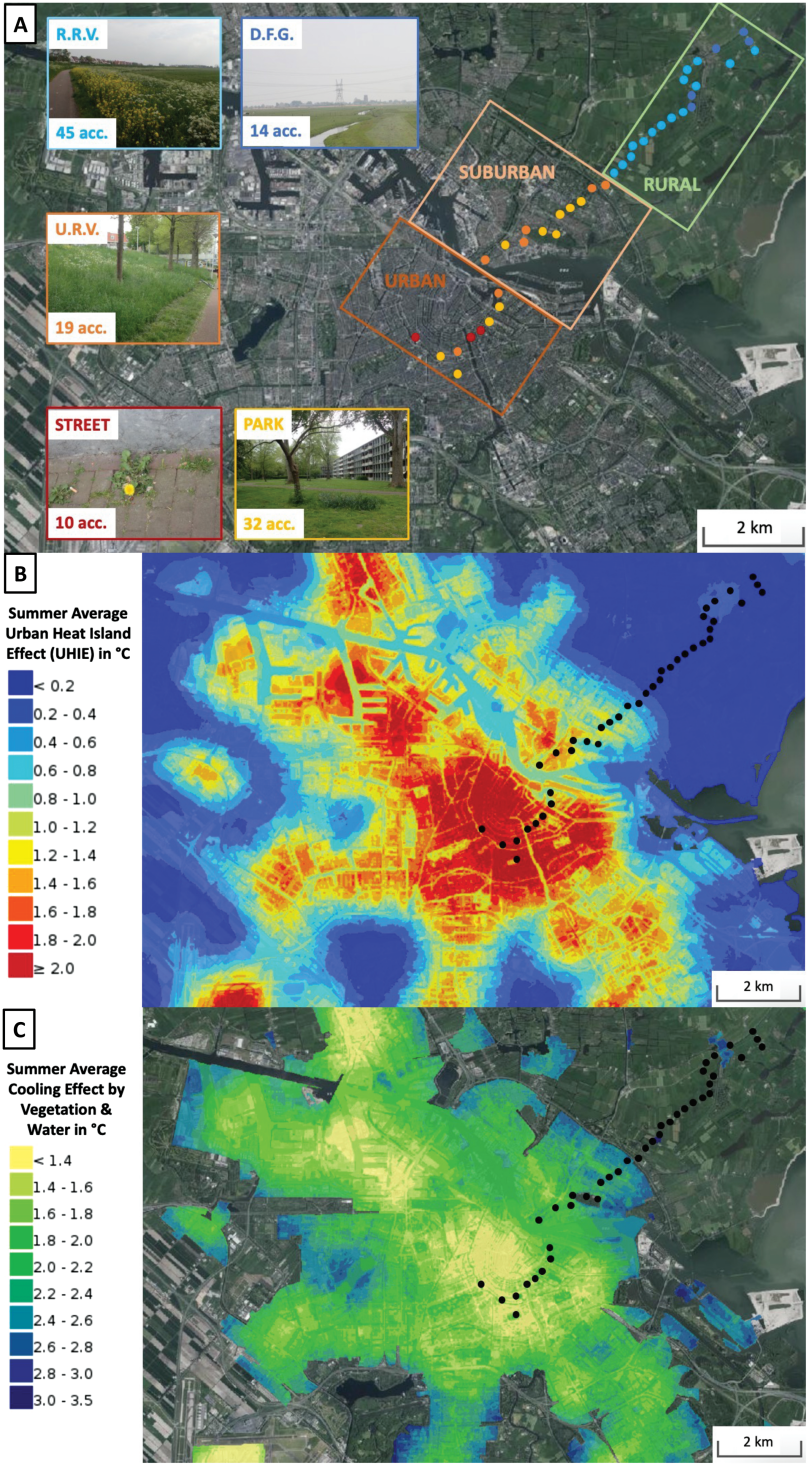
Overview of the Amsterdam (The Netherlands) urban–rural transect used in this study with the characterization of the urban heat island effect (UHIE). (A) Aerial photograph composition map of Amsterdam with the transect indicated by colored dots on the map. The transect is divided into three districts, according to the level of urbanization. Colors of dots correspond to the dominant subhabitat types (Amsterdam urban–rural transect for definitions) at each location, which are illustrated with example photographs: R.R.V. = rural roadside verge; U.R.V. = urban roadside verge; D.F.G. = dairy farm grassland. (B) Characterization of the UHIE imposed on the map from Figure 1A. Values are calculated on a 10 × 10 m resolution and correspond to the average temperature difference in the summer months (June–August). The map was built with a model that calculates the maximum UHIE based on population density and wind speed at 10 m height, which is subsequently corrected by the amount of impervious surface, vegetation, and water in a 1km radius. A final correction is based on a more localized effect of vegetation in a 30 m radius. The map reflects averages and daily values can be much higher (up to +10 °C, Van der Hoeven & Wandl, 2013). (C) Characterization of the cooling effect of vegetation and water, used to correct the maximum UHIE to obtain the average UHIE displayed in Figure 1B. Values are calculated on a 10 × 10 m resolution. All maps obtained from “Atlas Leefomgeving,” published by the Rijksinstituut voor Volksgezondheid en Milieu (RIVM, 2022) on 14 February 2023, https://www.atlasleefomgeving.nl/.

The common dandelion (*Taraxacum officinale* F.H. Wigg. s.l.) is one of the most widespread plant species in temperate areas, including The Netherlands where it is native, and occurs prolifically both in rural areas around cities and within cities, where it functions as one of the most important food sources for insects (Hicks et al., 2016). This species comprises both sexual and derived asexual (apomictic) individuals, where new apomictic genotypes arise repeatedly in mixed sexual-apomictic populations (Van Dijk et al., 2009). The city of Amsterdam lies in a geographic area where sexual reproduction is absent (Menken et al., 1995), but where the diversity of genotypes is high due to a constant influx of new clonal lineages (Van Der Hulst et al., 2003). Such diverse clonal assemblages can show highly repeatable (Lachapelle & Colegrave, 2017) and rapid adaptive responses to selection (Moerman & Colegrave, 2022), producing population-level adaptation by environmental filtering of (clonal) genotypes (Mazumder & Kesseli, 2021; McLeod et al., 2012; Schmitz et al., 2023), coupled with possible effects of de novo mutations occurring within clonal lineages. For instance, populations of apomictic dandelions display large genotypic variation in flowering phenology (Collier & Rogstad, 2004; Mazumder & Kesseli, 2021), providing a genetic reservoir for phenological adaptation. Apomixis also allows for easy replication of genotypes for phenotypic evaluations in common garden experiments, to test whether observed phenotypic divergence between urban and rural plants has a genetic basis. The above reasons make the common dandelion an ideal model for experimental studies on urban adaptation in plants.

The characteristics of cities that are most impactful on vegetation are increased temperatures, impervious surface cover, habitat fragmentation, changes in herbivore communities, and light, air, and soil pollution (Johnson & Munshi-South, 2017). Habitat fragmentation was found to alter dispersal characteristics in urban plants of holy hawksbeard (*Crepis sancta*) (Cheptou et al., 2008) and urban–rural adaptation to changes in herbivory pressure has been demonstrated in white clover (*Trifolium repens*) (Santangelo et al., 2022b) as well as dandelions (Pisman et al., 2020). More generally speaking, the combined pressures of the urban environment (pan-urban effects) have led to phenological adaptation in pepperweed (*Lepidium virginicum*) (Yakub & Tiffin, 2016) and common ragweed (*Ambrosia artemisiifolia*) (Gorton et al., 2018), and changes in life-history traits in *Arabidopsis thaliana* (Schmitz et al., 2023). Thermal differences between urban and rural areas have been studied in the form of lower urban winter soil temperatures (due to reduced snow cover) and found to decrease hydrogen cyanide production in white clover (Santangelo et al., 2020; Thompson et al., 2016). So far, plant adaptation to increased urban temperatures has only been demonstrated in the leaf color of a short-lived annual plant species (Fukano et al., 2023).

Here, we present further experimental evidence for a native long-lived perennial plant species adapting to the UHIE, demonstrating adaptive evolution in temperature-dependent seedling growth and flowering phenology in the common dandelion along an urban–rural transect. Specifically, we evaluated growth response to elevated temperatures and flowering response to different lengths of vernalization period. We did this in controlled common garden experiments using clonal seeds collected along an urban–rural transect in the city of Amsterdam, The Netherlands, an area that rapidly urbanized with a steep increase in stony (impervious) surfaces as compared to wooden houses between 1,600 and 1,650 (Abrahamse, 2010), generating conditions for adaptation to the UHIE. To study the spatial scale at which adaptation occurs along this urban–rural cline, we analyzed phenotypic divergence between plants in relation to (sub)urban vs. rural areas, subhabitats within the urban–rural mosaic (street, urban roadside verge, park, rural roadside verge, and dairy farm grassland), and distance to the center. We hypothesized that urban plants grow better at elevated temperatures and require shorter vernalization periods compared to rural plants. We show that adaptation to the UHIE occurs in dandelions, indicating that natural selection on diverse clonal assemblages can facilitate plant resilience to increased urban temperatures.

## Materials and methods

### Amsterdam urban–rural transect

We sampled common dandelion plants (*T. officinale* s.l. F.H. Wigg.) along an urban–rural gradient in Amsterdam, The Netherlands, following the protocol from the GLobal Urban Evolution project (GLUE, https://www.globalurbanevolution.com/). Briefly, GLUE transects were designed for sampling white clover (*Trifolium repens* L.) and are defined as (1) covering a continuous urbanization gradient defined by impervious surface cover (e.g., buildings and roads) decreasing with distance from downtown urban centers, (2) containing an equal amount of distance and number of sampling locations within and outside the urban area, and (3) contain a minimum total of 40 sampling locations with a minimum distance of 200 m between sampling locations. Due to the natural habitat of white clover, a high proportion of sampling locations occur within urban green spaces (parks and mowed lawns). We have therefore used the 40 locations in the original GLUE transect as a general guideline and have collected dandelion plants on varying degrees of the impervious substrate to include a wider variety of subhabitats present along the urban–rural transect. The Amsterdam transect (Figure 1) has 40 sampling locations and runs from the approximate geographical center of the city (sampling location 1: Sarphati Park, Oud-Zuid) through suburban areas (Amsterdam-Noord) to a small rural village (sampling location 40: Broek in Waterland), measuring a total distance of 12.03 km. In the late spring season (22–26 April 2020) we collected dandelion seed heads from three plants at each sampling location, aiming for a minimum of 50 m distance between accessions. In total, seed heads were collected from 120 accessions (40 sampling locations with 3 individuals per location).

To study differentiation among urban and rural dandelions at different spatial scales, we annotated the accessions collected along the transect in three different ways. First, we divided the transect into three general districts (Figure 1A and B) comprising urban (locations 1–9), suburban (locations 10–20), and rural (locations 21–40) districts. The distinction between urban and suburban districts was made based on age of the urbanization (where the urban center is several centuries older, Abrahamse, 2010), the severity of the UHIE (Figure 1B), and the strong geographical separation by water (Amsterdamse IJ). Although some variation in temperature exists within these districts, they do form rather distinct sub islands of UHIE (Figure 1B). Second, we defined subhabitats (Figure 1A) occurring along the urban–rural transect based on our own observations during the dandelion flowering season: (1) street/pavement—a continuous impervious surface (≥75% in a 5 m circle around the plant) of man-made structure (i.e., a road); (2) urban roadside verge—a green strip (planted border or lawn) next to an urban road (25–75% impervious surface cover); (3) city park—a continuous green zone (≤25% impervious surface cover) within the (sub)urban perimeter where car traffic is excluded; (4) rural roadside verge—a green strip of (naturalized) vegetation along the rural road (or bicycle lane); (5) dairy farm grassland—continuous zone of grassland (0% impervious surface cover) vegetation [usually a monoculture of English ryegrass (*Lolium perenne* L.) with a few wildflowers] that is grazed by dairy cattle or frequently mown for hay harvesting and typically highly fertilized. Finally, we calculated the distance for each accession to the start of the transect (sampling location 1) as an approximation of the distance from the center of the heat island (Figure 1B). Lacking detailed temperature measurements from reliable sources at each location of our transect, we used the distance metric as a proxy for differences in urban heat. We recorded the GPS locations in the field during collection using a smartphone (Motorola Moto G5, Motorola Inc., Chicago, IL) and measured distance in a straight line to the start of the transect using an online calculator (https://www.gps-coordinates.net/distance). An overview of categorical divisions for all accessions can be found in the online repository along with the data (Data and Code Availability Statement). Limited stock of clonal seeds is available for all accessions upon reasonable request with the corresponding authors.

To restrict our analyses to *T. officinale* s.l. (following Kirschner & Štěpánek, 2011), we filtered our set of accessions from the transect based on morphological characters observed in the greenhouse, as some diagnostic features can be more easily ascertained in flowering plants than in seeding plants without flowers, which is the typical stage of plants during seed collecting in the field. We used the position of outer involucral bracts in the mature inflorescence morphology as a diagnostic character (Kirschner & Štěpánek, 2011), marking those with dispersed or strongly reflexed bracts as *T. officinale* s.l. This selection was performed with the inflorescences obtained in the long vernalization experiment as nearly all plants reached flowering here. Ten accessions were thereby omitted from further analysis. One of these belonged to an urban street accession, one was collected along a suburban roadside verge and three in suburban parks. Among the five excluded rural accessions, three were collected along rural roadside verges and two in dairy farm grasslands (Supplementary Material).

We conducted all phenotyping experiments with plants in randomized order. Randomization was achieved by shuffling the list accessions in a Microsoft Excel sheet (using the function RAND to generate random values between 0 and 1 in a separate column and sorting the list on ascending values). This was repeated for each treatment and plants were placed on greenhouse benches or climate chamber blocks accordingly.

### Vernalization experiments

To determine differential vernalization requirements between urban and rural dandelions we performed two experiments in which we subjected plants from all accessions from the transect to three different lengths of a continuous cold period (4 °C): a long vernalization period of 109 days, a short period of 36 days and an absence of vernalization (control). Dandelion genotypes show large variation in vernalization requirements, ranging from no vernalization to several months (Sterk & Luteijn, 1984). A vernalization period of 2–3 months is generally cited for dandelions (Tas & Van Dijk, 1999; Van Baarlen et al., 2000; Van Dijk et al., 2003) and we therefore applied an excess of this in the long vernalization treatment (109 days) with the intention to satisfy vernalization requirements for all genotypes. According to weather data from Amsterdam Schiphol Airport, which is comparable in predicted heat island effects to central Amsterdam (RIVM, 2022), the average daily temperature drops below 4 °C for 13–43 days only (KNMI, 2024; data from 2019 to 2023). Thus, the 36-day vernalization treatment is comparable to natural vernalization as experienced by urban dandelions. Flowering time was recorded in days from the time of reentering the greenhouse after vernalization (Day 0) until the emergence of the first yellow petals from the first inflorescence. To apply consistency between vernalization experiments, plants that had not flowered after 56 days were scored as non-flowering (for details, see Supplementary Material Part 1). We considered not flowering as a lack of sufficient vernalization. Due to unsuccessful germinations, we had to exclude 5 accessions from the long vernalization experiment, 11 from the short vernalization treatment, and 8 from the control treatment (Supplementary Material).

#### Long vernalization experiment

Seeds collected in the field were germinated on agar plates for 11 days (20 °C, 16 hr light/16 °C 8 hr dark) in ECD01 incubators (Snijders Labs, Tilburg, The Netherlands). For each accession, one seedling was transplanted into a 9.3 × 9.3 × 10 cm pot containing, volume-wise, 50% potting soil and 50% fine sand (which are established growing conditions for greenhouse experiments with mature dandelions). Seedlings were then propagated in the greenhouse for 28 days and watered once or twice per week, as required. Subsequently, all plants were given a vernalization treatment by putting them in a cold chamber for 109 days (4 °C, 16 hr light/8 hr dark), watering occasionally as required to prevent wilting. Plants were then placed back into the greenhouse in random order, with watering every 2–3 days. Once a week we supplied a 2× diluted Hoagland nutrient solution (Hewitt, 1966) for optimal growth.

#### Short vs. no vernalization experiment

In a second vernalization experiment, a shortened cold treatment (36 days: 4 °C, 16 hr light/8 hr dark) was applied, as well as a no-vernalization treatment. Field-collected seeds were newly germinated (two per accession) and propagated from seedlings to small plants, using the conditions described in Long vernalization experiment, with the exception that a substrate mixture of 80% potting soil and 20% pumice was used. One batch of plants was subjected to the short vernalization treatment (36 days), while the other batch remained in the greenhouse to serve as a control for the absence of vernalization. Plants were placed on greenhouse benches in random order within treatments. Water and fertilizer supply followed the protocol described in Long vernalization experiment, supplemented by a singular supply of 1.3 g Osmocote® Exact Mini 5-6M slow-release fertilization pellets (ICL Group Ltd, Tel-Aviv, Israel) per pot.

### Heat experiment

For each flowering plant in the long vernalization experiment (Long vernalization experiment), the first developing inflorescence was used to produce clonal seeds for the heat experiment. Any potential cross-pollination was prevented by putting small paper bags over the inflorescences as soon as they opened. Bags were removed after 3–4 days when seeds started to develop. This can be seen as confirmation of apomixis, considering that common dandelions are self-incompatible (Meirmans et al., 2006), and therefore self-pollination can be discounted as a potential mode of fertilization. Due to the unsuccessful flowering of 18 accessions, these did not yield greenhouse-grown clonal seeds and therefore were excluded from the heat treatment experiment.

To test whether urban and rural dandelions differ in their growth response to elevated temperatures, we measured seedling biomass accumulation in urban and rural dandelions at a range of different temperatures. The seedling stage is part of the life cycle where dandelions naturally experience the most severe consequences of the UHIE: In the northern hemisphere temperate zone, seeds are dispersed in late May (when temperatures reach 15–25 °C), germinating upon soil contact and reaching seedling stage from late June, when daytime temperatures can increase with ≥8 °C (Van der Hoeven & Wandl, 2013) compared to rural areas. Temperatures of 26–32 °C are therefore normal conditions for urban dandelion seedlings to grow in. By using seeds that were generated in a common greenhouse environment, we minimize environmental maternal effects on seed quality as a source of phenotypic variation in experimental plants.

Seed germination and seedling propagation were performed using the conditions described in Long vernalization experiment, with the exception that smaller pots (7 × 7 × 8 cm) and a shorter seedling propagation time (7 days) were used. The length of the first non-cotyledon leaf (third leaf) was measured as an estimator of plant size at the beginning of the temperature treatment. Using MC 1750VHO climate cabinets (Snijders Labs), we grew the seedlings at 20, 26, 32, and 38 °C for 11 days (60% humidity, 16 hr light/8 hr dark). For each temperature, we used three replicate blocks with one plant from each accession placed in randomized order, leading to a total of three replicates for each accession. Because only two climate cabinets were available at one time, the replicates were split up in time slots. First, two temperature treatments were done for one replicate, whereafter the other two temperature treatments were performed. The second and third replicate blocks were then performed in the same manner. For this reason, seed germination and seedling propagation was performed in batches, to ensure equal germination propagation time for all plants in all temperature treatments. Plants were harvested after the temperature treatment, the roots were washed, and the whole plant was dried (70 °C, 1 week) before weighing the dry biomass.

### Statistical analysis

We used three different analysis approaches to test if the probability of flowering differed between plants according to their collection position along the transect. The three approaches capture the UHIE in different ways (Amsterdam urban–rural transect) to reveal at which scale adaptation is occurring. First, we used logistic regressions using distance from the start of the transect as a continuous variable to test if the flowering response showed a gradual change along the transect. Logistic regressions were performed using PROC GENMOD (SAS OnDemand for Academics, SAS Institute Inc., Cary, NC, USA) using Wald chi-square tests. Second, we used Fisher’s exact tests (PROC FREQ, SAS OnDemand for Academics) to test if the proportion of plants that flowered in our vernalization experiments differed between the three main transect districts: urban, suburban, and rural. Third, we used Fisher’s exact tests to test if the proportion of plants that flowered in our vernalization experiments differed between the five subhabitats: street, urban roadside verge, park, rural roadside verge, and dairy farm grassland. For the second experiment involving the short vernalization and control treatments, we fitted separate statistical models to data for each of the two treatment levels, because the vernalization treatment not only induces flowering capacity but also causes a delay in plant development, which makes it difficult to compare flowering dates between plants from the different treatments. However, we also analyzed a single model containing both short and no vernalization treatment levels, which is useful for evaluating the interaction effect between vernalization and different levels of urbanization (PROC GENMOD, SAS OnDemand for Academics, Supplementary Material Part 3).

For the heat experiment, we used linear mixed models to test the effects of temperature, collection position, and their interaction, on total plant biomass. In all models, we included replicate block and temperature treatment as categorical fixed factors and third leaf length at the start of the temperature treatment as a continuous fixed cofactor. In all models, we included “collection location of the accession” and its interaction with temperature treatment as fixed factors, where three separate models used different definitions of “collection location” to reveal the scale at which adaptation occurs: (1) distance from the start position of the transect (continuous cofactor); (2) main transect district (urban, suburban or rural; categorical factor); and (3) subhabitat type (street, urban roadside verge, park, rural roadside verge and dairy farm grassland; categorical factor). Linear models were performed using PROC GLM (SAS OnDemand for Academics). After plotting temperature treatment results as a function of distance to the start of the transect, we visualized trends in growth response at different temperatures along the urban–rural transect by fitting the regression line for each temperature treatment as estimated from a linear model as described above, when fitted to data from each temperature treatment separately. All *p*-values reported in this manuscript correspond to two-sided statistical testing.

## Results

We used seeds from dandelions collected along an urban–rural transect in and around Amsterdam (Figure 1) to analyze two traits in which we expected to find evidence of adaptation to the urban heat island: vernalization requirement (length of cold period necessary to induce flowering) and growth response to elevated temperatures (biomass accumulation).

### Flowering response to shortened vernalization periods

**Figure 2.**
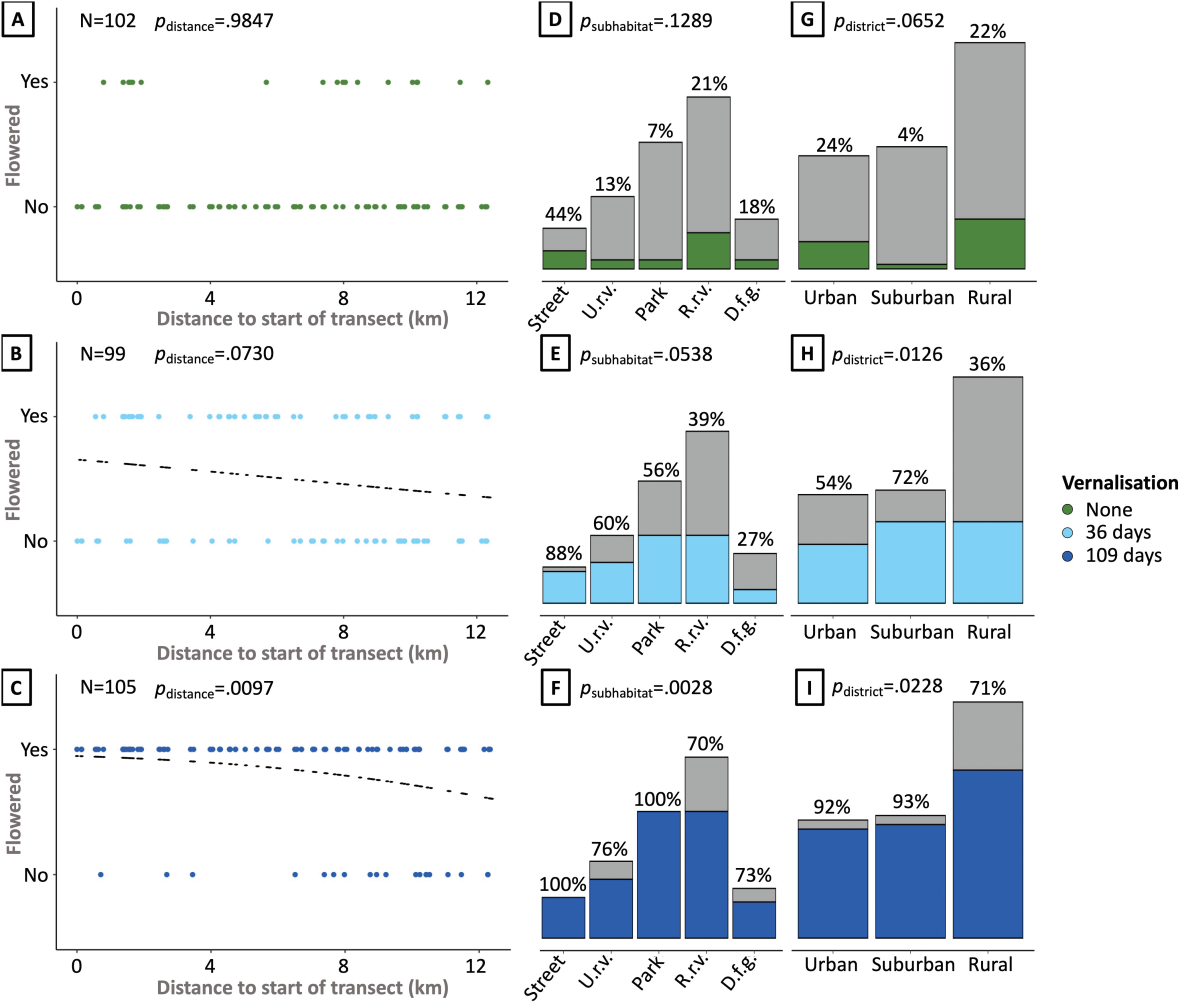
Flowering response to different vernalization treatments of plants from the urban–rural transect. Flowering response is visualized on three different scales in the urban–rural transect: for individual accessions with different distance to the start of the urban–rural transect—as response variable (A–C), per subhabitat (D–F, Amsterdam urban–rural transect for definitions where U.r.v. and R.r.v. stand for urban and rural roadside verge respectively, and D.f.g. stands for dairy farm grassland), and per district (G–I, Figure 1 for definitions). For bar plots (D–I), the proportion of flowering plants is visualized as the colored part of the bar, with gray corresponding to non-flowering plants. Small black dots in panels B and C indicate the model-predicted probability of flowering at each analyzed distance to the center of the UHI, as determined by logistic regression (Statistical analysis for details). *P*-values (“*p*=”) correspond to the effect of the indicated variable on flowering in the corresponding treatment and are calculated by logistic regressions (A–C) or Fisher’s exact tests (D–I). *N* indicates the number of samples in each treatment.

Rural plants had a higher vernalization requirement than urban plants, as exposed under a short (Figure 2H) and long (Figure 2C, F, and I) vernalization treatments. The absence of vernalization did not expose differences at any scale of urbanization (Figure 2, A, D, and G), whereas a short vernalization exposed significant (*p* = .0126) differences between urban, suburban, and rural districts (Figure 2H). Under a long vernalization, this differential requirement was even visible at finer scales of subhabitats (Figure 2F, *p* = .0028) and distance from the start of the transect (Figure 2C, *p* = .0097). Only 18 plants flowered in the absence of a vernalization treatment, irrespective of the sampling location of the accessions, however, a short vernalization treatment was sufficient to induce flowering in many of the plants from the urban (54%) and suburban districts (72%) but not of the rural plants (36%, Figure 2H). The interaction between short or no vernalization treatment and district was also significant (treatment × district effect *p* = .0013, Supplementary Material Part 2). Nearly all urban (92%) and suburban (93%) plants flowered after long vernalization (Figure 2I), whereas 29% of the rural plants still did not reach flowering (*p* = .0228, Figure 2I). The probability of flowering following long vernalization decreased with distance to the start of the transect (*p* = .0097, black dotted line in Figure 2C). We conclude that our vernalization treatments were long enough to satisfy the vernalization requirement for nearly all urban accessions, but not for all rural accessions.

For plants that did flower, no significant flowering time differences were observed between urban and rural plants when vernalization was applied (Supplementary Material Part 3). Urban district plants flowered slightly later than rural plants when no vernalization was applied. This was mainly the effect of earlier flowering in rural roadside verge plants in the absence of vernalization. We observed a significant difference between subhabitats in flowering response to long vernalization (*p* = .0028) and a close to significant response to short vernalization (*p* = .0538), where a >sevenfold increase was observed in flowering city park plants (Figure 2E). Under long vernalization, all street and park plants flowered (Figure 2F). Nearly all street plants flowered already after a short vernalization (from 4/9 in the absence of vernalization (Figure 2D) to 7/8 after short vernalization (Figure 2E) and to 9/9 after standard vernalization (Figure 2F)), whereas plants found in urban roadside verges (from 2/16 to 9/15 to 13/17, respectively) and city parks (from 2/28 to 15/27 to 28/28, respectively) required a long vernalization to reach similar proportions. The response was lowest in dairy farm grassland plants where only one additional plant was found flowering after a short vernalization treatment (Figure 2D and E). The proportion of non-flowering plants was highest among rural roadside verges, with 12 out of 40 plants failing to flower even after a long vernalization treatment (Figure 2F).

### Growth response to elevated temperatures

**Figure 3.**
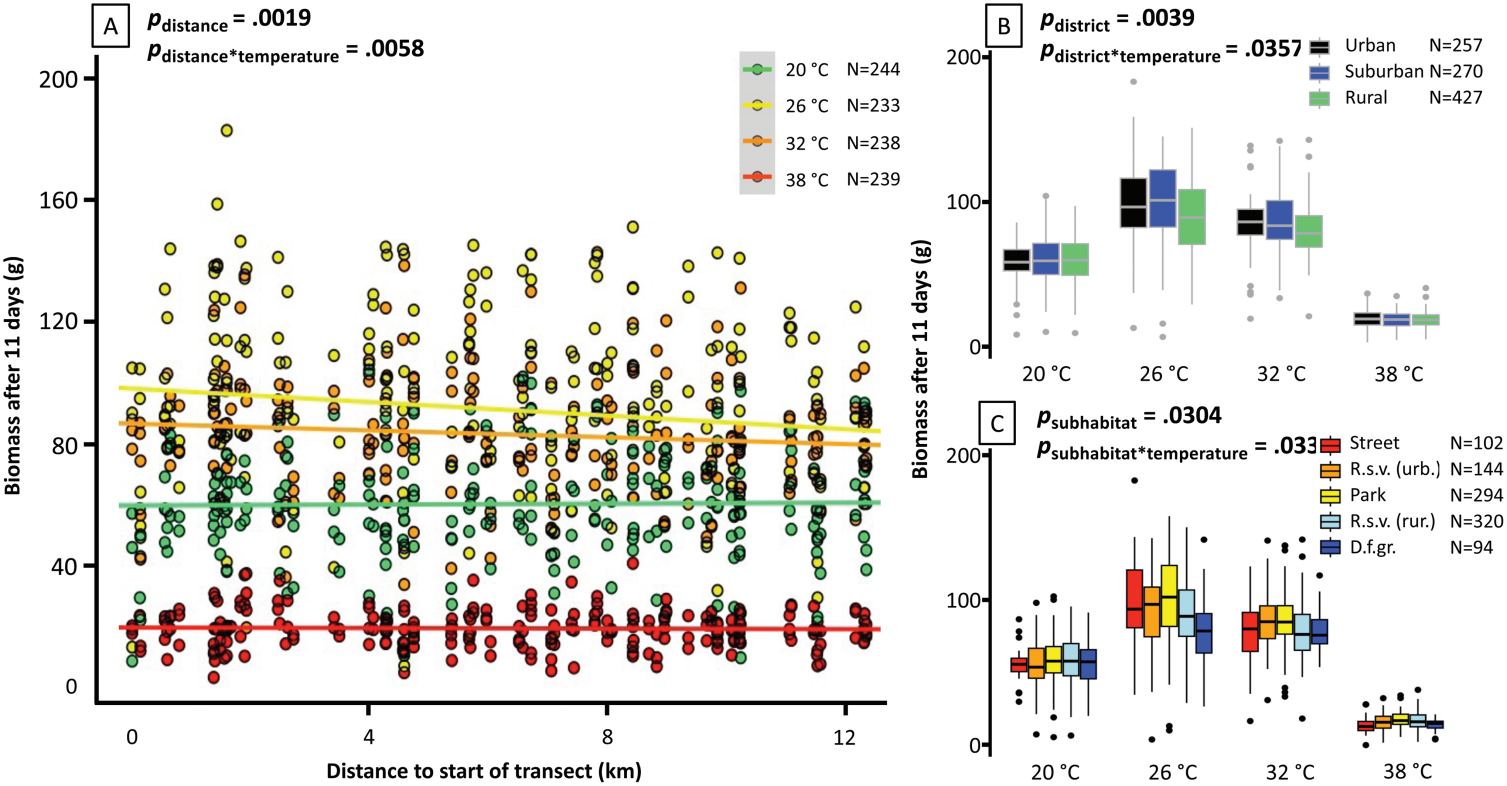
Growth of dandelion seedling plants (28 days after germination) at different elevated temperatures. All accessions collected along the Amsterdam transect were subjected to three elevated temperatures, and a control temperature (20 °C). Accumulated biomass was measured after 11 days of treatment as the dry weight of the plants and is visualized in relation to three spatial scales: (A) distance to the start of the urban–rural transect with colored lines indicating the linear model fit for biomass at the indicated temperature (Supplementary Material Part 5); (B) urban, suburban and rural districts; (C) subhabitats defined within the transect (Figure 1C–G), where “R.s.v.” abbreviates “roadside verge” (urban and rural) and “D.f.gr.” abbreviates “dairy farm grassland.” Definitions of the different variables are detailed in Amsterdam urban–rural transect. Boxplot (B and C) definitions: center lines, median values; lower and upper hinges, first and third quartiles, respectively; whiskers, 1.5× interquartile range; individual points, outliers. *P*-values (“*p=*”) correspond to the statistical tests of the interaction between accessions and treatment in relation to the variable indicated (Table 1 for details). *N* corresponds to the number of samples in each treatment (A) or subcategorization (B and C).

Seedling growth response to elevated temperatures differed along the transect (Figure 3A). Increasing the growing temperature from 20 °C to 26 °C and 32 °C caused a significantly stronger growth increase in accessions collected close to the center of the UHI compared to accessions collected outside the city (temperature treatment × transect position interaction effect: *p* = .0058, Table 1 and Figure 3). This resulted in a higher biomass for urban and suburban plants compared to rural plants especially at 26 °C (Figure 3B), while higher biomass of urban compared to rural plants was not observed at 20 °C (Figure 3A).

**Table 1.** Statistical test results of the effects of different vernalization treatments on flowering success (see Fig. 2) and of temperature on seedling biomass accumulation (see Fig. 3). Distance from the start of the urban–rural transect is analyzed as a continuous variable, district, and habitat as categorical variables. ^Reported for the distance model, see Supplementary Material Part 5 for results of similar models that fit effects of subhabitats or districts instead of distance from the start of the transect.

Vernalization (Exp 1—Long)	Chi-square	dF	*p*
Distance from start transect	6.69	1	.0097
District	8.03	2	.0228
Subhabitat	12.96	4	.0028

Vernalization (Exp 2—Short)	Chi-square	dF	*p*
Distance from start transect	3.21	1	.0730
District	8.92	2	.0126
Subhabitat	9.38	4	.0538

Vernalization (Exp 2—None)	Chi-square	dF	*p*
Distance from start transect	0.00	1	.9847
District	4.96	2	.0652
Subhabitat	7.17	4	.1289

Heat treatment—Distance model	*F*-value	dF	*p*
Block	96.52	2; 931	<.0001
Initial leaf length	561.24	1; 931	<.0001
Temperature	332.13	3; 931	<.0001
Distance from start transect	9.73	1; 931	.0019
Temperature × Distance	4.20	3; 931	.0058

Heat treatment—District model	*F*-value	dF	*p*
Block	96.28	2; 927	<.0001
Initial leaf length	555.84	1; 927	<.0001
Temperature	1,042.02	3; 927	<.0001
District	5.58	2; 927	.0039
Temperature × District	2.26	6; 927	.0357

Heat treatment—Subhabitat model	*F*-value	dF	*p*
Block	93.49	2; 919	<.0001
Initial leaf length	541.51	1; 919	<.0001
Temperature	828.89	3; 919	<.0001
Subhabitat	2.68	4; 919	.0304
Temperature × Subhabitat	1.88	12; 919	.0337

Growth differences between subhabitats were negligible at 20 °C but became apparent at higher temperatures, especially at 26 °C, where plants from urban subhabitats attained higher biomass than plants from rural subhabitats (Supplementary Material Part 5 and Figure 3C). Accessions from rural dairy farm grasslands profited least from the temperature increase to 26 °C. Differences disappeared when increasing to 38 °C.

## Discussion

The UHIE is a globally consistent consequence of urbanization (Rizwan et al., 2008), considerably affecting human health (Heaviside et al., 2017; Shahmohamadi et al., 2011) as well as the co-inhabiting biodiversity (Johnson & Munshi-South, 2017). Given the high rate of global urbanization (Haase et al., 2018) and the mitigating effect that (even small) plants have on the UHIE by shading and covering impervious surfaces (Tan et al., 2021), it is relevant to understand the adaptive capacity of wild plant species to this novel environment. In addition to leaf color, as described by Fukano et al., (2023), this study presents further experimental evidence for plant adaptation to the UHIE in seedling growth and flowering phenology, using common dandelion as a model species for wild plant adaptation. We demonstrated adaptive divergence in growth response to increased temperatures using common garden experiments with dandelions from the city of Amsterdam, The Netherlands. Urban and suburban plants responded more strongly to increases in temperature than rural plants, by showing a larger increase in biomass accumulation (Figure 3B). In addition, we found differential vernalization requirements along the urban–rural gradient, where urban and suburban plants were more responsive to short vernalization than rural plants (Figure 2H). The UHIE leads to higher temperatures, especially in summer (Manoli et al., 2020), during the growing season of plants (Kabano et al., 2021) and shorter vernalization exposure due to milder winters (Rizwan et al., 2008). Our results therefore provide evidence of adaptation to the UHIE.

Proof of adaptation requires the demonstration of genetic differentiation as well as fitness gain (Lambert et al., 2021), meaning that divergence alone is not enough to claim adaptive evolution. Taking the biomass measurements in the heat treatment experiment as a proxy for plant fitness, we interpret our result of stronger growth response to increased temperatures in urban accessions as evidence for adaptation to urban heat, while we consider the reduced vernalization requirement in urban accessions as suggestive evidence for adaptation to mild urban winters. First, by using common garden experimental designs for phenotypic analysis, genetic differentiation is demonstrated for both traits. Due to the apomictic clonal mode of reproduction in dandelions, the greenhouse-grown plants are genetically identical to the wild mother plant from which the seeds were collected. Conclusions from such common garden experiments are often complicated by the presence of maternal effects (Wolf & Wade, 2009), which are phenotypic effects in offspring that reflect the environmental conditions in which the maternal parent was growing, e.g., by means of seed quality, and not reflecting the offspring’s genotype. Differences between urban and rural plants grown in a common environment may therefore (partly) reflect differences between mother plants and their respective environmental plasticity (Wolf & Wade, 2009). Such effects can be discounted in the results from our heat response results as the experiment was conducted using clonal second-generation seeds harvested from greenhouse-grown plants, strengthening our evidence for adaptive evolution. Second, a fitness gain can be inferred from the heat treatment results, as urban plants generated more biomass than rural plants in response to temperature increases. The ability to generate more biomass is strongly linked with higher fecundity in plants, making it a suitable proxy for fitness in adaptation studies (Younginger et al., 2017). Higher biomass production in urban plants was temperature-dependent, and only observed at higher temperatures reflecting urban growing conditions (26–32 °C), thus strengthening the conclusion that this adaptation is a response to the UHIE. Interestingly, our treatment of 38 °C seems to be suboptimal for all plants, even for those adapted to higher growing temperatures (Figure 3). The optimal growing temperature for (urban) dandelions seems to be at or slightly above 26 °C.

The fact that this response is adaptive through a superior growth rate suggests that the urban–rural gradient pattern has been shaped by natural selection. Our experimental design was aimed specifically to reveal the presence of adaptive responses to the UHIE temperature gradient. Such temperature adaptation can contribute to an overall pattern of local adaptation (Lambert et al., 2021) in urban versus rural dandelion populations. However, adaptation to other environmental differences than temperature remains to be demonstrated, and reciprocal transplant (Blanquart et al., 2013) or reciprocal common garden experiments (Lortie & Hierro, 2021) would be necessary to reveal if an overall pattern of local adaptation exists.

We interpret the lower vernalization requirement in urban plants (Figure 2), on the other hand, as tentative evidence for adaptation, because our experimental evidence alone does not directly demonstrate a performance or fitness advantage of the observed reduced vernalization requirement. Previous studies have demonstrated shifts in flowering phenology for the overall urban plant community in relation to the UHIE (Christmann et al., 2022; Li et al., 2019; Zipper et al., 2016) but have always relied on field observations in whole ecosystems. Such observations cannot distinguish between phenotypic plasticity and genetic divergence. Our study overcomes these limitations by demonstrating a differential response to shorter vernalization periods in a single plant species using a common environment. Overall, the urban dandelion population displays a lower vernalization requirement than the rural population, which is an expected result of selection in response to shorter and milder urban winters due to the UHIE. Such adaptation could result from the selective loss of genotypes (environmental filtering) with long vernalization requirements, which might not be satisfied in cities as winter surface temperatures are 1–2 °C higher due to the UHIE (Manoli et al., 2020). While our vernalization results are consistent with the hypothesis of adaptation to the UHIE, additional empirical evidence on the fitness benefits of shorter vernalization requirements in cities is desired to strengthen the adaptive interpretation of the observed genetic divergence in this trait. Additionally, it may be informative to follow the flowering response to different vernalization treatments over several years. Dandelions are long-lived perennial plants and may still reproduce in other years, despite not flowering in some years due to insufficient vernalization. Although a lost reproductive season will therefore not immediately lead to the termination of the respective genotype, the genotype’s lifetime fitness is considerably reduced by failing to reproduce in some years.

The urban environment is a complex mosaic of different habitats (Santangelo et al., 2022a), where completely impervious (such as roads and pavements) and very pervious habitats (such as parks) occur in proximity. Small changes in UHIE will occur between such different urban areas and a linear transect will not perfectly capture the decrease in UHIE from the city center to the rural area. With our definitions of subhabitats (Figure 1A), we were able to capture part of this environmental heterogeneity and revealed subtle differences in heritable traits between plants from these categories. In the vernalization treatments, urban street plants had the highest flowering rate of all subhabitats after short vernalization exposure (Figure 2B). The difference with urban roadside verges, which is also visible after long vernalization exposure (Figure 2C), might reflect differences in selection pressures for reduced vernalization. This may be due to a lower difference between day and night temperatures in urban habitats with high impervious surface density (Van der Hoeven & Wandl, 2013). Rural roadside verges, on the other hand, may reflect a slightly urbanized version of the countryside with roads presenting slightly higher local temperatures due to the presence of asphalt. This is suggested by the higher biomass accumulation of rural roadside verge plants at 26 °C compared to rural grassland plants (Figure 3C). Taken together, we recommend a denser sampling of different intra-urban and -rural subhabitats to further investigate adaptation to this aspect of urban habitats. Anthropogenic activities affecting plant traits measured here (biomass, flowering), such as mowing, sweeping, trampling, and farming, may also reflect urban environmental heterogeneity and should therefore be considered in future studies.

Having demonstrated adaptation to urban heat in apomictic dandelions, a relevant question is to what extent our results are generalizable to other plant species. As our experiment included accessions from only one city, and patterns of urban evolution can differ between cities (Santangelo et al., 2022b), it would be of particular interest to investigate the adaptive responses we discovered here in other cities of varying size. Although there can be significant structural differences between cities leading to different adaptation patterns (Santangelo et al., 2022a), the UHIE is a globally consistent pressure on wild flora and fauna (Rizwan et al., 2008) and adaptations to it may therefore be expected to be consistent between cities, as found in adaptive leaf color variation by Fukano et al. (2023).

We used the clonal mode of reproduction found in dandelions to our advantage here as it allowed us to establish a common environment experiment with genetically identical copies of the wild plants. Additionally, adaptation may be facilitated in apomictic dandelion populations as they form highly diverse clonal assemblages (Van Der Hulst et al., 2003). Such systems can respond rapidly to selection by environmental filtering of clones (Moerman & Colegrave, 2022) however, it is unclear if the long-term populations’ adaptive potential remains sufficient in these asexual assemblages. It would therefore be of interest to study urban heat adaptation in an outcrossing sexual species, in contrast with the clonal assemblages studied here. Clonality and asexual reproduction are predicted as favorable traits in urban environments due to the lower abundance and functional diversity of pollinators (Johnson et al., 2015; Theodorou et al., 2020; Van Drunen & Johnson, 2022) and the lower availability of nearby sexual mates. Diverse clonal assemblages such as dandelions may therefore, at least in the short term, have a competitive ecological advantage over sexually reproducing plants.

Successful adaptation to the UHIE, as we show here, may also have broader implications for adaptations to climate change. Continued global warming and increased frequency of extreme weather events, such as intensified heat waves, are predicted for the coming decades (Pörtner et al., 2022) but are already normalized conditions in urban environments (Manoli et al., 2020; Rizwan et al., 2008). The UHIE might therefore act as a selective pressure toward preadaptation to climate change (Diamond & Martin, 2021). It would be interesting to further investigate whether plant populations from warmer native climates have a larger fitness in urban heat islands (Čeplová et al., 2017; Exposito-Alonso, 2023; Géron et al., 2021). Furthermore, global warming will likely increase the UHIE significantly in the near future, requiring further adaptation to heat. Facilitating UHIE adaptation of naturally occurring flora, for instance via increased connectivity between natural urban and rural areas, could therefore have a positive effect on future UHIE mitigation and on native plant adaptation to climate change in general.

## Supplementary material

Supplementary material is available online at *Evolution Letters*.

qrae040_suppl_Supplementary_Material

## Data Availability

Data and code supporting the results reported in this manuscript are available at the open-access digital repository Figshare, under DOI: 10.6084/m9.figshare.23567259. Additional results on flowering time response to different vernalization treatments are described in Supplementary material file.

## References

[CIT0001] Abrahamse, J. E. (2010). De grote uitleg van Amsterdam: Stadsontwikkeling in de zeventiende eeuw [PhD thesis]. Faculty of Humanities (FGw), University of Amsterdam. https://dare.uva.nl/search?identifier=2b6e3e43-c68e-4d79-95d3-b2375865c55f

[CIT0002] Blanquart, F., Kaltz, O., Nuismer, S. L., & Gandon, S. (2013). A practical guide to measuring local adaptation. Ecology Letters, 16(9), 1195–1205. https://doi.org/10.1111/ele.12150 23848550

[CIT0003] Brans, K. I., Jansen, M., Vanoverbeke, J., Tüzün, N., Stoks, R., & De Meester, L. (2017). The heat is on: Genetic adaptation to urbanization mediated by thermal tolerance and body size. Global Change Biology, 23(12), 5218–5227. https://doi.org/10.1111/gcb.13784 28614592

[CIT0004] Campbell-Staton, S. C., Winchell, K. M., Rochette, N. C., Fredette, J., Maayan, I., Schweizer, R. M., & Catchen, J. (2020). Parallel selection on thermal physiology facilitates repeated adaptation of city lizards to urban heat islands. Nature Ecology and Evolution, 4(4), 652–658. https://doi.org/10.1038/s41559-020-1131-8 32152530

[CIT0005] Čeplová, N., Kalusová, V., & Lososová, Z. (2017). Effects of settlement size, urban heat island and habitat type on urban plant biodiversity. Landscape and Urban Planning, 159, 15–22. https://doi.org/10.1016/j.landurbplan.2016.11.004

[CIT0006] Cheptou, P. -O., Carrue, O., Rouifed, S., & Cantarel, A. (2008). Rapid evolution of seed dispersal in an urban environment in the weed *Crepis sancta*. Proceedings of the National Academy of Sciences of the United States of America, 105(10), 3796–3799. https://doi.org/10.1073/pnas.0708446105 18316722 PMC2268839

[CIT0007] Christmann, T., Kowarik, I., Bernard-Verdier, M., Buchholz, S., Hiller, A., Seitz, B., & von der Lippe, M. (2022). Phenology of grassland plants responds to urbanization. Urban Ecosystem, 26(1), 261–275. https://doi.org/10.1007/s11252-022-01302-y

[CIT0008] Collier, M. H., & Rogstad, S. H. (2004). Clonal variation in floral stage timing in the common dandelion *Taraxacum officinale* (Asteraceae). American Journal of Botany, 91(11), 1828–1833. https://doi.org/10.3732/ajb.91.11.1828 21652330

[CIT0009] Diamond, S. E., Chick, L. D., Perez, A., Strickler, S. A., & Martin, R. A. (2018). Evolution of thermal tolerance and its fitness consequences: parallel and non-parallel responses to urban heat islands across three cities. Proceedings Biological Sciences, 285(1882), 20180036. https://doi.org/10.1098/rspb.2018.0036 30051828 PMC6053939

[CIT0010] Diamond, S. E., & Martin, R. A. (2021). Physiological adaptation to cities as a proxy to forecast global-scale responses in climate change. Journal of Experimental Biology, 224(Pt suppl 1), jeb229336. https://doi.org/10.1242/jeb.229336 33627462

[CIT0011] Exposito-Alonso, M. (2023). Understanding local plant extinctions before it is too late: Bridging evolutionary genomics with global ecology. New Phytologist, 237(6), 2005–2011. https://doi.org/10.1111/nph.18718 36604850

[CIT0012] Fukano, Y., Yamori, W., Misu, H., Sato, M. P., Shirasawa, K., Tachiki, Y., & Uchida, K. (2023). From green to red: Urban heat stress drives leaf color evolution. Science Advances, 9(42), eabq3542. https://doi.org/10.1126/sciadv.abq3542 37862418 PMC10588939

[CIT0013] Géron, C., Lembrechts, J. J., Borgelt, J., Lenoir, J., Hamdi, R., Mahy, G., Nijs, I., & Monty, A. (2021). Urban alien plants in temperate oceanic regions of Europe originate from warmer native ranges. Biological Invasions, 23(6), 1765–1779. https://doi.org/10.1007/s10530-021-02469-9

[CIT0014] Gorton, A. J., Moeller, D. A., & Tiffin, P. (2018). Little plant, big city: A test of adaptation to urban environments in common ragweed (*Ambrosia artemisiifolia*). Proceedings Biological Sciences, 285(1881), 20180968. https://doi.org/10.1098/rspb.2018.0968 30051853 PMC6030533

[CIT0015] Haase, D., Güneralp, B., Dahiya, B., Bai, X., & Elmqvist, T. (2018). Global urbanization. In: T. Elmqvist, X. Bai, N. Frantzeskaki, C. Griffith, D. Maddox, T. McPhearson, S. Parnell, P. Romero-Lankao, D. Simon, & M. Watkins (Eds.), Urban planet: Knowledge towards sustainable cities (pp. 326–339). Cambridge University Press.

[CIT0016] Heaviside, C., Macintyre, H., & Vardoulakis, S. (2017). The urban heat Island: Implications for health in a changing environment. Current Environmental Health Reports, 4(3), 296–305. https://doi.org/10.1007/s40572-017-0150-3 28695487

[CIT0017] Hewitt, E. J. (1966). Sand and water culture methods used in the study of plant nutrition. Technical communication / Commonwealth Bureau of Horticulture and Plantation Crops; no. 22 (Rev. 2nd ed.). Commonwealth Agricultural Bureaux, Farnham Royal, Bucks.

[CIT0018] Hicks, D. M., Ouvrard, P., Baldock, K. C. R., Baude, M., Goddard, M. A., Kunin, W. E., Mitschunas, N., Memmott, J., Morse, H., Nikolitsi, M., Osgathorpe, L. M., Potts, S. G., Robertson, K. M., Scott, A. V., Sinclair, F., Westbury, D. B., & Stone, G. N. (2016). Food for pollinators: Quantifying the nectar and pollen resources of urban flower meadows. PLoS One, 11(6), e0158117. https://doi.org/10.1371/journal.pone.0158117 27341588 PMC4920406

[CIT0019] Johnson, M. T. J., & Munshi-South, J. (2017). Evolution of life in urban environments. Science, 358(6363), eaam8327. https://doi.org/10.1126/science.aam8327 29097520

[CIT0020] Johnson, M. T. J., Thompson, K. A., & Saini, H. S. (2015). Plant evolution in the urban jungle. American Journal of Botany, 102(12), 1951–1953. https://doi.org/10.3732/ajb.1500386 26620097

[CIT0021] Kabano, P., Lindley, S., & Harris, A. (2021). Evidence of urban heat island impacts on the vegetation growing season length in a tropical city. Landscape and Urban Planning, 206, 103989. https://doi.org/10.1016/j.landurbplan.2020.103989

[CIT0022] Kerstes, N. A. G., Breeschoten, T., Kalkman, V. J., & Schilthuizen, M. (2019). Snail shell colour evolution in urban heat islands detected via citizen science. Communications Biology, 2, 264. https://doi.org/10.1038/s42003-019-0511-6 31341963 PMC6642149

[CIT0023] Kirschner, J., & Štěpánek, J. (2011). Typification of *Leontodon taraxacum* L. (≡ *Taraxacum officinale* F.H. Wigg.) and the generic name Taraxacum: A review and a new typification proposal. TAXON, 60(1), 216–220. https://doi.org/10.1002/tax.601021

[CIT0024] Koninklijk Nederlands Meteorologisch Instituut (KNMI). (2024). *Archive of daily weather measurements for The Netherlands*. https://daggegevens.knmi.nl/klimatologie/daggegevens; accessed on 17 June 2024.

[CIT0025] Lachapelle, J., & Colegrave, N. (2017). The effect of sex on the repeatability of evolution in different environments. Evolution, 71(4), 1075–1087. https://doi.org/10.1111/evo.13198 28181234

[CIT0026] Lambert, M. R., Brans, K. I., Des Roches, S., Donihue, C. M., & Diamond, S. E. (2021). Adaptive evolution in cities: Progress and misconceptions. Trends in Ecology and Evolution, 36(3), 239–257. https://doi.org/10.1016/j.tree.2020.11.002 33342595

[CIT0027] Li, D., Stucky, B. J., Deck, J., Baiser, B., & Guralnick, R. P. (2019). The effect of urbanization on plant phenology depends on regional temperature. Nature Ecology and Evolution, 3(12), 1661–1667. https://doi.org/10.1038/s41559-019-1004-1 31712691

[CIT0028] Lortie, C. J., & Hierro, J. L. (2021). A synthesis of local adaptation to climate through reciprocal common gardens. Journal of Ecology, 110, 1015–1021.

[CIT0029] Manoli, G., Fatichi, S., Bou-Zeid, E., & Katul, G. G. (2020). Seasonal hysteresis of surface urban heat islands. Proceedings of the National Academy of Sciences of the United States of America, 117(13), 7082–7089. https://doi.org/10.1073/pnas.1917554117 32184330 PMC7132285

[CIT0030] Mazumder, L., & Kesseli, R. (2021). Population structure, seasonal genotypic differentiation, and clonal diversity of weedy dandelions in three Boston area populations (*Taraxacum* sp.). Ecology and Evolution, 11(16), 10926–10935. https://doi.org/10.1002/ece3.7870 34429891 PMC8366855

[CIT0031] McLean, M. A., Angilletta, M. J., & Williams, K. S. (2005). If you can’t stand the heat, stay out of the city: Thermal reaction norms of chitinolytic fungi in an urban heat island. Journal of Thermal Biology, 30, 384–391.

[CIT0032] McLeod, K. A., Scascitelli, M., & Vellend, M. (2012). Detecting small-scale genotype–environment interactions in apomictic dandelion (*Taraxacum officinale*) populations. Journal of Evolutionary Biology, 25(8), 1667–1675. https://doi.org/10.1111/j.1420-9101.2012.02549.x 22694090

[CIT0033] Meirmans, P. G., Den Nijs, H. (j)C. M., & Van Tienderen, P. H. (2006). Male sterility in triploid dandelions: Asexual females vs asexual hermaphrodites. Heredity, 96, 45–52. 16189541 10.1038/sj.hdy.6800750

[CIT0034] Menken, S. B. J., Smit, E., & Nijs, H. (J. )C. M. D. (1995). Genetical population structure in plants: Gene flow between diploid sexual and triploid asexual dandelions (Taraxacum Section Ruderalia). Evolution, 49, 1108–1118. 28568531 10.1111/j.1558-5646.1995.tb04437.x

[CIT0035] Moerman, F., & Colegrave, N. (2022). Frequent sexual reproduction limits adaptation in outcrossed populations of the alga *Chlamydomonas reinhardtii*. biorXiv, preprint: not peer reviewed. Date Accessed 1 June 1, 2023. https://doi.org/10.1101/2022.07.01.498510

[CIT0036] Piano, E., De Wolf, K., Bona, F., Bonte, D., Bowler, D. E., Isaia, M., Lens, L., Merckx, T., Mertens, D., van Kerckvoorde, M., De Meester, L., & Hendrickx, F. (2017). Urbanization drives community shifts towards thermophilic and dispersive species at local and landscape scales. Global Change Biology, 23(7), 2554–2564. https://doi.org/10.1111/gcb.13606 27997069

[CIT0037] Pisman, M., Bonte, D., & de la Peña, E. (2020). Urbanization alters plastic responses in the common dandelion *Taraxacum officinale*. Ecology and Evolution, 10(9), 4082–4090. https://doi.org/10.1002/ece3.6176 32489632 PMC7244812

[CIT0038] Pörtner, H.-O., Roberts, D.C., Tignor, M.M.B., Poloczanska, E., Mintenbeck, K., & Alegría, A., Craig, M., Langsdorf, S., Löschke, S., et al. (2022). Climate change 2022: Impacts, adaptation and vulnerability. IPCC.

[CIT0040] Rijksinstituut voor Volksgezondheid & Milieu (RIVM). (2022). *Atlas Natuurlijk Kapitaal: Stedelijk hitte-eiland effect (UHI) in Nederland*. https://www.atlasleefomgeving.nl/; accessed on 13 February 2023.

[CIT0041] Rivkin, L. R., Santangelo, J. S., Alberti, M., Aronson, M. F. J., de Keyzer, C. W., Diamond, S. E., Fortin, M. -J., Frazee, L. J., Gorton, A. J., Hendry, A. P., Liu, Y., Losos, J. B., MacIvor, J. S., Martin, R. A., McDonnell, M. J., Miles, L. S., Munshi-South, J., Ness, R. W., Newman, A. E. M., … Johnson, M. T. J. (2019). A roadmap for urban evolutionary ecology. Evolutionary Applications, 12(3), 384–398. https://doi.org/10.1111/eva.12734 30828362 PMC6383741

[CIT0042] Rizwan, A. M., Dennis, L. Y. C., & Liu, C. (2008). A review on the generation, determination and mitigation of Urban Heat Island. Journal of Environmental Sciences, 20, 120–128. 10.1016/s1001-0742(08)60019-4 18572534

[CIT0043] Samach, A., & Coupland, G. (2000). Time measurement and the control of flowering in plants. BioEssays, 22, 38–47. 10649289 10.1002/(SICI)1521-1878(200001)22:1<38::AID-BIES8>3.0.CO;2-L

[CIT0044] Santangelo, J. S., Ness, R. W., Cohan, B., Fitzpatrick, C. R., Innes, S. G., Koch, S., Miles, L. S., Munim, S., Peres-Neto, P. R., Prashad, C., Tong, A. T., Aguirre, W. E., Akinwole, P. O., Alberti, M., Álvarez, J., Anderson, J. T., Anderson, J. J., Ando, Y., Andrew, N. R., … Johnson, M. T. J. (2022b). Global urban environmental change drives adaptation in white clover. Science, 375(6586), 1275–1281. https://doi.org/10.1126/science.abk0989 35298255

[CIT0045] Santangelo, J. S., Roux, C., & Johnson, M. T. J. (2022a). The effects of environmental heterogeneity within a city on the evolution of clines. Journal of Ecology, 110, 2950–2959.

[CIT0046] Santangelo, J. S., Thompson, K. A., Cohan, B., Syed, J., Ness, R. W., & Johnson, M. T. J. (2020). Predicting the strength of urban-rural clines in Mendelian polymorphism along a latitudinal gradient. Evolution Letters, 4, 212–225. 32547782 10.1002/evl3.163 PMC7293085

[CIT0047] Schmitz, G., Linstädter, A., Frank, A. S. K., Dittberner, H., Schrader, A., & Thome, J., et al. (2023). Environmental filtering of life-history trait diversity in urban populations of *Arabidopsis thaliana*. biorXivpreprint: not peer reviewed. Date Accessed on 1 June 2023. https://doi.org/10.1101/2022.10.03.510679

[CIT0048] Shahmohamadi, P., Che-Ani, A. I., Etessam, I., Maulud, K. N. A., & Tawil, N. M. (2011). Healthy environment: The need to mitigate urban heat island effects on human health. Procedia Engineering, 20, 61–70. https://doi.org/10.1016/j.proeng.2011.11.139

[CIT0049] Sterk, A. A., & Luteijn, M. M. (1984). A study of the flowering phenology of Taraxacum microspecies in some biotopes in The Netherlands as observed during three successive years. Acta Botanica Neerlandica, 33(1), 39–59. https://doi.org/10.1111/j.1438-8677.1984.tb01771.x

[CIT0050] Tan, J. K. N., Belcher, R. N., Tan, H. T. W., Menz, S., & Schroepfer, T. (2021). The urban heat island mitigation potential of vegetation depends on local surface type and shade. Urban Forestry & Urban Greening, 62, 127128. https://doi.org/10.1016/j.ufug.2021.127128

[CIT0051] Tas, I. C. Q., & Van Dijk, P. J. (1999). Crosses between sexual and apomictic dandelions (Taraxacum). I. The inheritance of apomixis. Heredity, 83 (Pt 6), 707–714. https://doi.org/10.1046/j.1365-2540.1999.00619.x 10651915

[CIT0052] Theodorou, P., Radzevičiūtė, R., Lentendu, G., Kahnt, B., Husemann, M., Bleidorn, C., Settele, J., Schweiger, O., Grosse, I., Wubet, T., Murray, T. E., & Paxton, R. J. (2020). Urban areas as hotspots for bees and pollination but not a panacea for all insects. Nature Communications, 11(1), 576. https://doi.org/10.1038/s41467-020-14496-6 PMC6989530 31996690

[CIT0053] Thompson, K. A., Renaudin, M., & Johnson, M. T. J. (2016). Urbanization drives the evolution of parallel clines in plant populations. Proceedings Biological Sciences, 283(1845), 20162180. https://doi.org/10.1098/rspb.2016.2180 28003451 PMC5204167

[CIT0054] Tüzün, N., Op de Beeck, L., Brans, K. I., Janssens, L., & Stoks, R. (2017). Microgeographic differentiation in thermal performance curves between rural and urban populations of an aquatic insect. Evolutionary Applications, 10(10), 1067–1075. https://doi.org/10.1111/eva.12512 29151861 PMC5680628

[CIT0055] Tzavali, A., Paravantis, J., Mihalakakou, G., Fotiadi, A., & Stigka, E. (2015). Urban heat island intensity: A literature review. Fresenius Environment Bulletin, 24, 4537–4554.

[CIT0057] Van Baarlen, P., Van Dijk, P. J., Hoekstra, R. F., & De Jong, J. H. (2000). Meiotic recombination in sexual diploid and apomictic triploid dandelions (*Taraxacum officinale* L.). Genome, 43(5), 827–835. https://doi.org/10.1139/g00-047 11081973

[CIT0058] Van Der Hoeven, F., & Wandl, A. (2013). Amsterwarm: Gebiedstypologie warmte-eiland. TU Delft.

[CIT0059] Van Der Hulst, R. G. M., Mes, T. H. M., Falque, M., Stam, P., Den Nijs, J. C. M., & Bachmann, K. (2003). Genetic structure of a population sample of apomictic dandelions. Heredity, 90(4), 326–335. https://doi.org/10.1038/sj.hdy.6800248 12692586

[CIT0060] Van Dijk, P., de Jong, H., Vijverberg, K., & Biere, A. (2009). An Apomixis-gene’s view on dandelions. In I. Schön, K. Martens, & P. Dijk (Eds.), Lost sex: The evolutionary biology of parthenogenesis (pp. 475–493). Springer Netherlands.

[CIT0061] Van Dijk, P. J., Van Baarlen, P., & De Jong, J. H. (2003). The occurrence of phenotypically complementary apomixis-recombinants in crosses between sexual and apomictic dandelions (*Taraxacum officinale*). Sexual Plant Reproduction, 16(2), 71–76. https://doi.org/10.1007/s00497-003-0177-5

[CIT0062] Van Drunen, W. E., & Johnson, M. T. J. (2022). Polyploidy in urban environments. Trends in Ecology and Evolution, 37(6), 507–516. https://doi.org/10.1016/j.tree.2022.02.005 35246321

[CIT0064] Wolf, J. B., & Wade, M. J. (2009). What are maternal effects (and what are they not)? Philosophical Transactions of the Royal Society of London, Series B: Biological Sciences, 364(1520), 1107–1115. https://doi.org/10.1098/rstb.2008.0238 19324615 PMC2666680

[CIT0065] Wong, N. H., Tan, C. L., Kolokotsa, D. D., & Takebayashi, H. (2021). Greenery as a mitigation and adaptation strategy to urban heat. Nature Reviews Earth & Environment, 2(3), 166–181. https://doi.org/10.1038/s43017-020-00129-5

[CIT0066] Yakub, M., & Tiffin, P. (2016). Living in the city: Urban environments shape the evolution of a native annual plant. Global Change Biology, 23(5), 2082–2089. https://doi.org/10.1111/gcb.13528 27718531

[CIT0067] Younginger, B. S., Sirová, D., Cruzan, M. B., & Ballhorn, D. J. (2017). Is biomass a reliable estimate of plant fitness? Applications in Plant Sciences, 5(2), 1600094. 10.3732/apps.1600094 PMC5315378 28224055

[CIT0068] Zipper, S. C., Schatz, J., Singh, A., Kucharik, C. J., Townsend, P. A., & Loheide, S. P. (2016). Urban heat island impacts on plant phenology: Intra-urban variability and response to land cover. Environmental Research Letters, 11, 054023.

