# Supplementary material for: Some like it hot: adaptation to the urban heat island in common dandelion
Source: Evol Lett. 2024 Jul 31;8(6):881–92. doi: 10.1093/evlett/qrae040 (PMC11637554; doi:10.1093/evlett/qrae040)
Supplement: qrae040_suppl_Supplementary_Material [file qrae040_suppl_supplementary_material.pdf]

Supporting Information to the manuscript "Some like it hot: adaptation to the urban heat island in common dandelion." in *Evolution Letters*.

Yannick Woudstra, Ron Kraaiveld, Alger Jorritsma, Kitty Vijverberg, Slavica Ivanovic, Roy Erkens, Heidrun Huber, Barbara Gravendeel, Koen J.F. Verhoeven

Contents:

Part 1 - Overview of &melines for vernalisa&on experiments
Part 2 - Statistics describing the interaction between vernalisa&on and urbanisation
Part 3 - Flowering time results
Part 4 - Detailed statistics of the vernalisation experiments
Part 5 - Detailed statistics of the heat trement experiment

Supporting Information – Part 1: Overview of timelines for vernalisation experiments

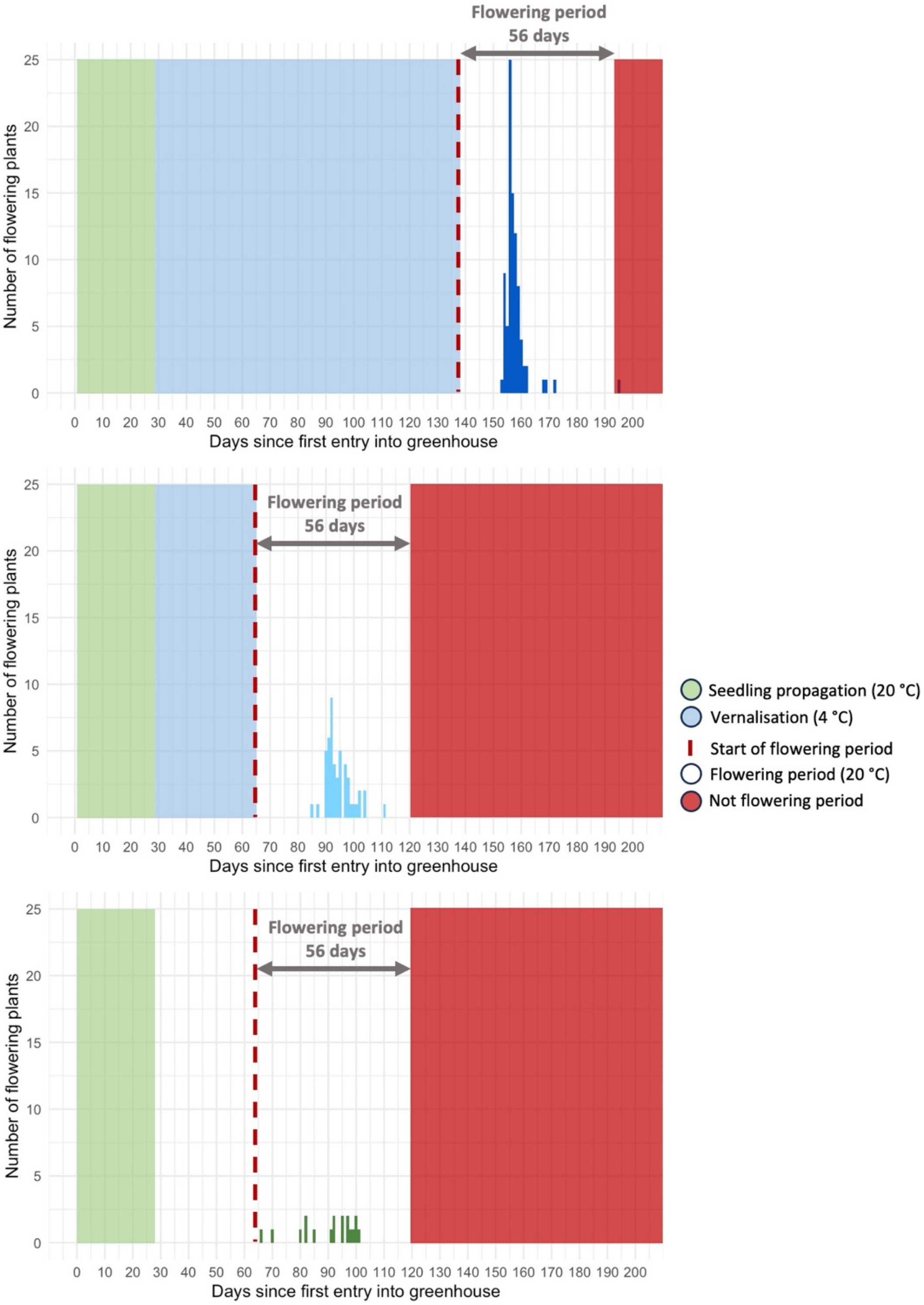

Figure S1.1: Overview of the timelines for the vernalisation experiments. All experiments started with a 10-day seed germination followed by a 28-day growing period in the greenhouse (green highlight). Day 0 marks the end of the germination and the start of the greenhouse growing period. The long vernalisation experiment (top panel) was first performed and comprised a 109-day vernalisation in climate chambers, after which flowering response was recorded for 107 days. The short vernalisation treatment (middle panel) and control (bottom panel) were performed as one experiment, in which vernalised plants spent 36 days in the cold climate chambers and control plants remained in the greenhouse. Flowering response was measured for 56 days after re-entry of the vernalised plants into the greenhouse. To compare flowering time results, this was measured from the day vernalisation ended. For analyses, the same allowed flowering period was chosen in the long vernalisation experiment, leading to the correction of one accession (which flowered after 58 days) to non-flowering. For control plants, there was no vernalisation and flowering time was therefore recorded from the moment vernalised plants re-entered the greenhouse.

Supporting Information – Part 2 – Statistics describing the interaction between vernalisation and urbanization

Table S2.1: Results of statistical tests (GLM Procedure) investigating the effect of the interaction between vernalisation treatment ('Treatment', 36 days vs none) and distance to the centre of the urban heat island ('Distance', defined as the start of the urban-rural transect) on flowering response.

**Log likelihood**	**Full log likelihood**	**AIC**	**AICC**	**BIC**			
-114.4929	-114.4929	236.9857	237.1898	250.1989			
**Parameter**	**Degrees of Freedom**	**Estimate**	**Standard Error**	**Likelihood Ratio 95% Confidence Limits**		**Wald Chi-Square**	**Pr > Chi-Square**
**Intercept**	1	0.6403	0.4217	-0.1736	1.4925	2.31	0.1289
**Distance**	1	-0.0001	0.0001	-0.0002	0	3.21	0.0730
**Treatment – 0 d**	1	-2.1896	0.6774	-3.5808	-0.9078	10.45	0.0012
**Treatment – 36 d**	0	0	0	0	0	.	.
**Distance*Treatment – 0 d**	1	0.0001	0.0001	-0.0001	0.0003	1.28	0.2580
**Distance*Treatment – 36 d**	0	0	0	0	0	.	.
**Scale**	0	1	0	1	1		
**Source**	**Degrees of Freedom**	**Chi-Square**	**Pr > Chi-Square**				
**Distance**	1	3.31	0.0688				
**Treatment**	1	11.64	0.0006				
**Distance*Treatment**	1	1.29	0.2563				

Table S2.2: Results of statistical tests (GLM Procedure) investigating the effect of the interaction between vernalisation treatment ('Treatment', 36 days vs none) and district (urban, suburban or rural) on flowering response.

**Log likelihood**	**Full log likelihood**	**AIC**	**AICC**	**BIC**			
-108.4463	-108.4463	228.8927	229.3256	248.7125			
**Parameter**	**Degrees of Freedom**	**Estimate**	**Standard Error**	**Likelihood Ratio 95% Confidence Limits**		**Wald Chi-Square**	**Pr > Chi-Square**
**Intercept**	1	-0.5754	0.2946	-1.1726	-0.0095	3.81	0.0508
**District – Urban**	1	0.7424	0.5046	-0.2421	1.7494	2.16	0.1412
**District – Suburban**	1	1.5198	0.5341	0.5076	2.6212	8.10	0.0044
**District – Rural**	0	0	0	0	0	.	.
**Treatment – 0 d**	1	-0.6903	0.4509	-1.5978	0.1819	2.34	0.1258
**Treatment – 36 d**	0	0	0	0	0	.	.
**District – Urban *Treatment – 0 d**	1	-0.6294	0.7684	-2.1690	0.8612	0.67	0.4127
**District – Suburban *Treatment – 0 d**	1	-3.5123	1.2001	-6.5839	-1.4699	8.57	0.0034
**District – Rural *Treatment – 0 d**	0	0	0	0	0	.	.
**District – Urban *Treatment – 36 d**	0	0	0	0	0	.	.
**District – Suburban *Treatment – 36 d**	0	0	0	0	0	.	.
**District – Rural *Treatment – 36 d**	0	0	0	0	0	.	.
**Scale**	0	1	0	1	1		
**Source**	**Degrees of Freedom**	**Chi-Square**	**Pr > Chi-Square**				
**District**	1	31.55	<.0001				
**Treatment**	1	1.69	0.4302				
**District*Treatment**	1	13.24	0.0013				

Table S2.3: Results of statistical tests (GLM Procedure) investigating the effect of the interaction between vernalisation treatment ('Treatment', 36 days vs none) and subhabitat (Methods section 1 for definitions) on flowering response.

**Log likelihood**	**Full log likelihood**	**AIC**	**AICC**	**BIC**			
-107.7822	-107.7822	235.5644	236.7223	268.5974			
**Parameter**	**Degrees of Freedom**	**Estimate**	**Standard Error**	**Likelihood Ratio 95% Confidence Limits**		**Wald Chi-Square**	**Pr > Chi-Square**
**Intercept**	1	-0.9808	0.6770	-2.4993	0.2597	2.10	0.1474
**Subhabitat – Street**	1	2.9267	1.2654	0.7464	6.0852	5.35	0.0207
**Subhabitat – Urban roadside verge**	1	1.3863	0.8580	-0.2288	3.2037	2.61	0.1061
**Subhabitat – Park**	1	1.2040	0.7800	-0.2551	2.8825	2.38	0.1227
**Subhabitat – Rural roadside verge**	1	0.5534	0.7540	-0.8564	2.1870	0.54	0.4630
**Subhabitat – Dairy farm grassland**	0	0	0	0	0	.	.
**Treatment – 0 d**	1	-0.5232	1.0341	-2.7368	1.5000	0.26	0.6129
**Treatment – 36 d**	0	0	0	0	0	.	.
**Subhabitat – Street *Treatment – 0 d**	1	-1.6458	1.6317	-5.2625	1.4684	1.02	0.3131
**Subhabitat – Urban roadside verge *Treatment – 0 d**	1	-1.8281	1.3852	-4.6525	0.9253	1.74	0.1869
**Subhabitat – Park *Treatment – 0 d**	1	-2.2648	1.3259	-4.9813	0.3817	2.92	0.0876
**Subhabitat – Rural roadside verge *Treatment – 0 d**	1	-0.3711	1.1567	-2.6350	2.0382	0.10	0.7484
**Subhabitat – Dairy farm grassland *Treatment – 0 d**	0	0	0	0	0	.	.
**Subhabitat – Street *Treatment – 36 d**	0	0	0	0	0	.	.
**Subhabitat – Urban roadside verge *Treatment – 36 d**	0	0	0	0	0	.	.
**Subhabitat – Park *Treatment – 36 d**	0	0	0	0	0	.	.
**Subhabitat – Rural roadside verge *Treatment – 36 d**	0	0	0	0	0	.	.
**Subhabitat – Dairy farm grassland *Treatment – 36 d**	0	0	0	0	0	.	.
**Scale**	0	1	0	1	1		
**Source**	**Degrees of Freedom**	**Chi-Square**	**Pr > Chi-Square**				
**District**	1	19.64	<.0001				
**Treatment**	1	10.51	0.0327				
**Subhabitat*Treatment**	1	6.20	0.1847				

Table S2.4: Results of statistical tests (GLM Procedure) investigating the effect of the interaction between vernalisation treatment (‘Treatment’, 36 days vs none) and distance to the centre of the urban heat island (‘Distance’, defined as the start of the urban-rural transect) on flowering time. ^: measured in days from the end of the vernalisation (36 days) treatment (Supporting Information Part 1 for details).

**Source**	**Degrees of Freedom**	**Sum of Squares**	**Mean Square**	**F-value**	**Pr > F**
**Model**	3	808.390336	269.463445	7.06	0.0004
**Error**	63	2404.027575	38.159168		
**Corrected Total**	66	3212.417910			
**R-Square**	**Coefficient Variation**	**Root Mean Square Error**	**Flowering Time^ Mean**		
0.251645	21.07331	6.177311	29.31343		
**Source**	**Degrees of Freedom**	**Sum of Squares (Type I)**	**Mean Square**	**F-value**	**Pr > F**
Distance	1	179.6331028	179.6331028	4.71	0.0338
Treatment	1	220.1198482	220.1198482	5.77	0.0193
Distance*Treatment	1	408.6373848	408.6373848	10.71	0.0017
**Source**	**Degrees of Freedom**	**Sum of Squares (Type III)**	**Mean Square**	**F-value**	**Pr > F**
Distance	1	353.5012398	353.5012398	9.26	0.0034
Treatment	1	95.9192433	95.9192433	2.51	0.1179
Distance*Treatment	1	408.6373848	408.6373848	10.71	0.0017

Table S2.5: Results of statistical tests (GLM Procedure) investigating the effect of the interaction between vernalisation treatment (‘Treatment’, 36 days vs none) and district (urban, suburban or rural) on flowering time. ^: measured in days from the end of the vernalisation (36 days) treatment (Supporting Information Part 1 for details).

**Source**	**Degrees of Freedom**	**Sum of Squares**	**Mean Square**	**F-value**	**Pr > F**
**Model**	5	956.033684	191.206737	5.17	0.0005
**Error**	61	2256.384227	36.989905		
**Corrected Total**	66	3212.417910			
**R-Square**	**Coefficient Variation**	**Root Mean Square Error**	**Flowering Time^ Mean**		
0.297606	20.74794	6.081933	29.31343		
**Source**	**Degrees of Freedom**	**Sum of Squares (Type I)**	**Mean Square**	**F-value**	**Pr > F**
District	2	343.0458596	171.5229298	4.64	0.0133
Treatment	1	152.7339512	152.7339512	4.13	0.0465
District*Treatment	2	460.2538727	230.1269364	6.22	0.0035
**Source**	**Degrees of Freedom**	**Sum of Squares (Type III)**	**Mean Square**	**F-value**	**Pr > F**
District	2	500.6268767	250.3134384	6.77	0.0022
Treatment	1	0.3334117	0.3334117	0.01	0.9247
District*Treatment	2	460.2538727	230.1269364	6.22	0.0035

Table S2.6: Results of statistical tests (GLM Procedure) investigating the effect of the interaction between vernalisation treatment ('Treatment', 36 days vs none) and subhabitat (Methods section 1 for definitions) on flowering time. ^: measured in days from the end of the vernalisation (36 days) treatment (Supporting Information Part 1 for details).

**Source**	**Degrees of Freedom**	**Sum of Squares**	**Mean Square**	**F-value**	**Pr > F**
**Model**	9	1315.208783	146.134309	4.39	0.0002
**Error**	57	1897.209127	33.284371		
**Corrected Total**	66	3212.417910			
**R-Square**	**Coefficient Variation**	**Root Mean Square Error**	**Flowering Time^ Mean**		
0.409414	19.68129	5.769261	29.31343		
**Source**	**Degrees of Freedom**	**Sum of Squares (Type I)**	**Mean Square**	**F-value**	**Pr > F**
Subhabitat	4	530.9668061	132.7417015	3.99	0.0064
Treatment	1	168.9235017	168.9235017	5.08	0.0281
Subhabitat*Treatment	4	615.3184758	153.8296189	4.62	0.0027
**Source**	**Degrees of Freedom**	**Sum of Squares (Type III)**	**Mean Square**	**F-value**	**Pr > F**
Subhabitat	4	844.2113396	211.0528349	6.34	0.0003
Treatment	1	20.0667686	20.0667686	0.60	0.4407
Subhabitat*Treatment	4	615.3184758	153.8296189	4.62	0.0027

Supporting Information – Part 3: Flowering time results

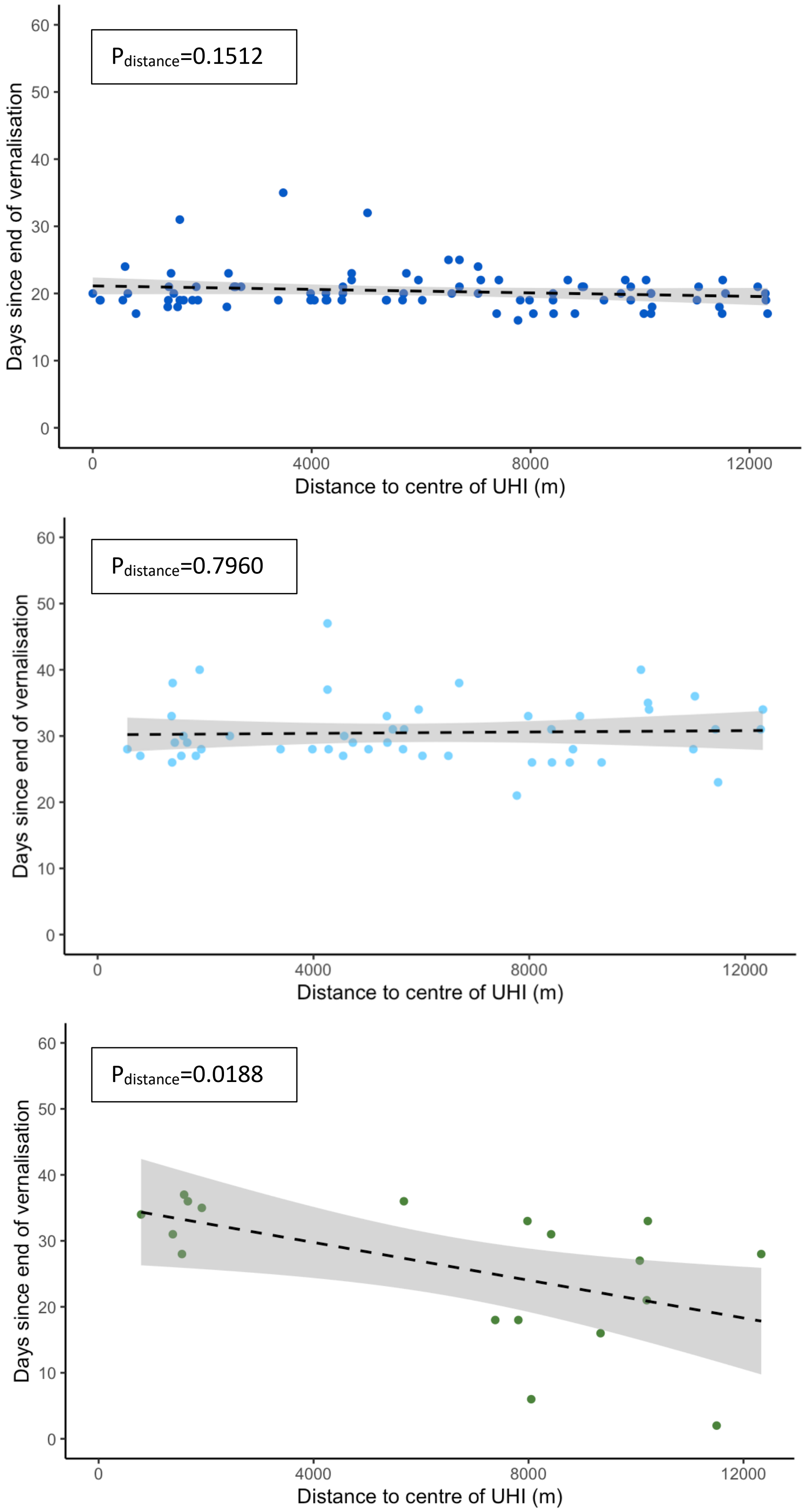

Figure S3.1: Flowering time results for plants collected along the urban-rural transect after different vernalization periods. A long (standard) vernalisation of 109 days (top panel, dark blue) was performed in the first experiment. In the second experiment, a shortened vernalisation of 36 days (middle panel, light blue) was applied as well as a control where no vernalisation was applied (bottom panel, green). For control plants, the end of the vernalisation treatment is defined as the end of the short vernalization treatment. P-values correspond to the effect of distance to the centre of the urban heat island (UHI) – defined as the start of the urban-rural transect – on flowering time in the corresponding treatment and are calculated by generalised linear models. Dashed lines indicate mean values as fitted by a linear model, with the grey shaded areas indicating standard error.

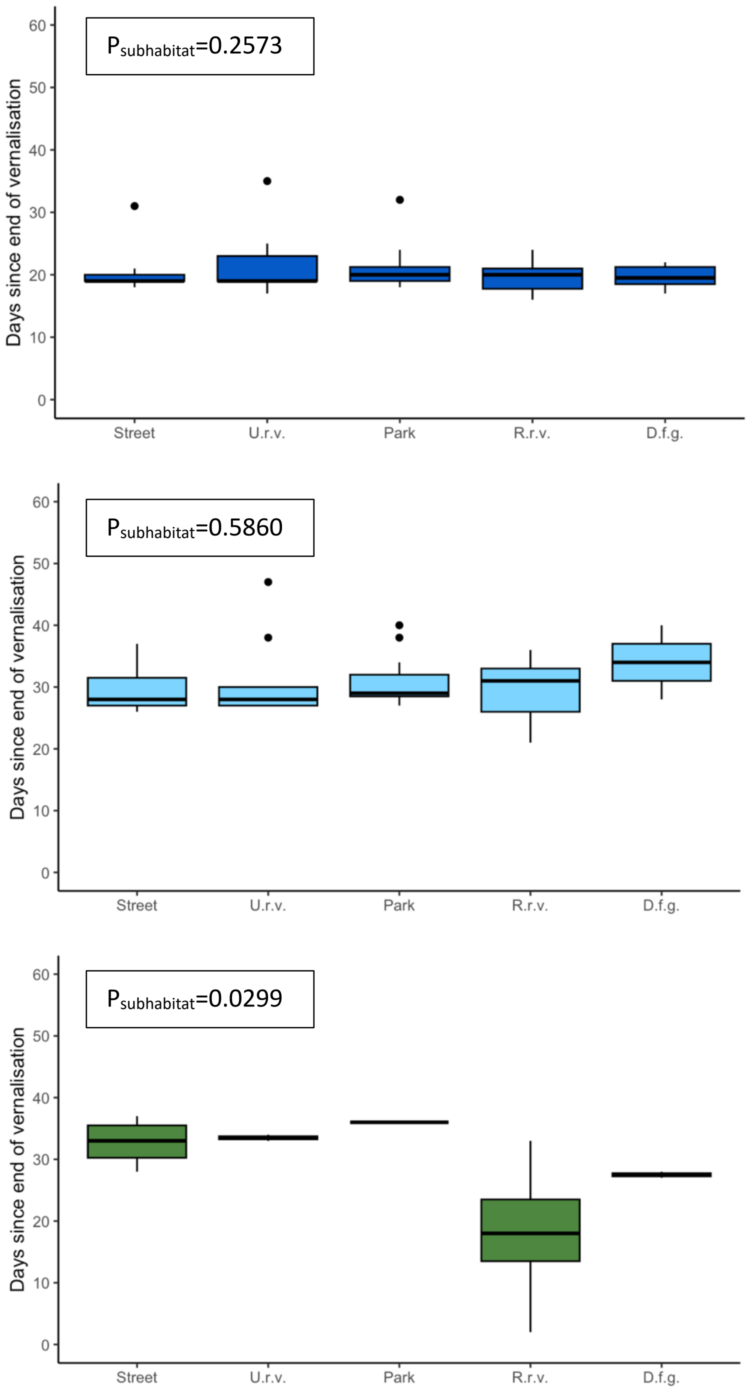

Figure S3.2: Flowering time results per subhabitat after different vernalization periods. A long (standard) vernalisation of 109 days (top panel, dark blue) was performed in the first experiment. In the second experiment, a shortened vernalisation of 36 days (middle panel, light blue) was applied as well as a control where no vernalisation was applied (bottom panel, green). For control plants, the end of the vernalisation treatment is defined as the end of the short vernalization treatment. Abbreviations: U.r.v. = urban roadside verge, R.r.v.= rural roadside verge, D.f.g. = dairy farm grassland. Boxplot definitions: centre lines, median values; lower and upper hinges, first and third quartiles, respectively; whiskers, 1.5x interquartile range; individual points, outliers. P-values correspond to the effect of subhabitat to the centre of the urban heat island (UHI) – defined as the start of the urban-rural transect – on flowering time in the corresponding treatment and are calculated by generalised linear models.

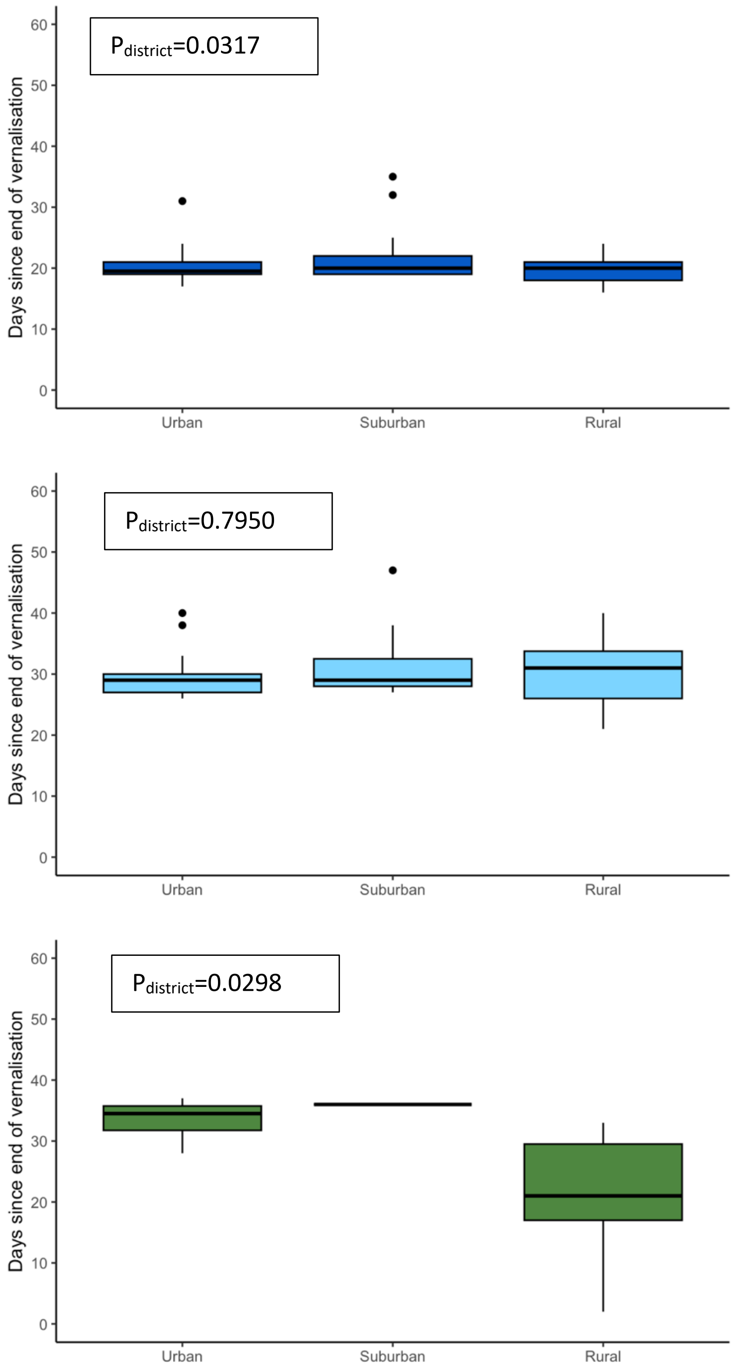

Figure S3.3: Flowering time results per district after different vernalization periods. A long (standard) vernalisation of 109 days (top panel, dark blue) was performed in the first experiment. In the second experiment, a shortened vernalisation of 36 days (middle panel, light blue) was applied as well as a control where no vernalisation was applied (bottom panel, green). For control plants, the end of the vernalisation treatment is defined as the end of the short vernalization treatment. Boxplot definitions: centre lines, median values; lower and upper hinges, first and third quartiles, respectively; whiskers, 1.5x interquartile range; individual points, outliers. P-values correspond to the effect of district to the centre of the urban heat island (UHI) – defined as the start of the urban-rural transect – on flowering time in the corresponding treatment and are calculated by generalised linear models.

Table S4.1: Results of the statistical analyses for the two vernalisation experiments. Distance to the centre of the urban heat island (UHI) – defined as the start of the urban-rural transect – was analysed as a continuous variable in a logistic regression. Subhabitat and district (Methods section 1 for definitions) were analysed as categorical variables in a Fisher's exact test.

**Long vernalisation (109 days)**							
**Distance to centre of UHI**							
**Log likelihood**	**AIC**	**AICC**	**BIC**				
-45.7105	95.4210	95.5386	100.7289				
**Parameter**	**Degrees of Freedom**	**Estimate**	**Standard Error**	**Likelihood Ratio 95% Confidence Limits**		**Wald Chi-Square**	**Pr > ChiSq**
**Intercept**	1	3.0629	0.7162	1.8076	4.6562	18.29	<.0001
**Distance**	1	-0.0002	0.0001	-0.0004	-0.0001	6.69	.0097
**Scale**	0	1.0000	0.0000	1.0000	1.0000		
**Subhabitat**				**District**			
	**Degrees of Freedom**	**Value**	**Probability**		**Degrees of Freedom**	**Value**	**Probability**
**Chi-Square**	4	12.9632	.0115	**Chi-Square**	2	8.0348	.0180
**Likelihood Ratio Chi-Square**	4	18.9849	.0008	**Likelihood Ratio Chi-Square**	2	8.4547	.0146
**Mantel-Haenszel Chi-Square**	1	8.0662	.0045	**Mantel-Haenszel Chi-Square**	1	6.4261	.0112
**Phi coefficient**	-	0.3514	-	**Phi coefficient**	-	0.2766	-
**Contingency coefficient**	-	0.3315	-	**Contingency coefficient**	-	0.2666	-
**Cramer's V**	-	0.3514	-	**Cramer's V**	-	0.2766	-
**Table Probability**	-	-	<.0001	**Table Probability**	-	-	.0014
**Pr <= P**	-	-	.0028	**Pr <= P**	-	-	.0228
**Short vernalisation (36 days)**							
**Distance to centre of UHI**							
**Log likelihood**	**AIC**	**AICC**	**BIC**				
-66.9611	137.9222	138.0472	143.1125				
**Parameter**	**Degrees of Freedom**	**Estimate**	**Standard Error**	**Likelihood Ratio 95% Confidence Limits**		**Wald Chi-Square**	**Pr > ChiSq**
**Intercept**	1	0.6403	0.4217	-0.1736	1.4925	2.31	.1289
**Distance**	1	-0.0001	0.0001	-0.0002	0.0000	3.21	.0730
**Scale**	0	1.0000	0.0000	1.0000	1.0000		
**Subhabitat**				**District**			
	**Degrees of Freedom**	**Value**	**Probability**		**Degrees of Freedom**	**Value**	**Probability**
**Chi-Square**	4	9.3811	.0522	**Chi-Square**	2	8.9175	.0116
**Likelihood Ratio Chi-Square**	4	10.0452	.0397	**Likelihood Ratio Chi-Square**	2	9.1394	.0104
**Mantel-Haenszel Chi-Square**	1	3.5585	.0592	**Mantel-Haenszel Chi-Square**	1	3.6502	.0561
**Phi coefficient**	-	0.3078	-	**Phi coefficient**	-	0.3001	-
**Contingency coefficient**	-	0.2942	-	**Contingency coefficient**	-	0.2875	-
**Cramer's V**	-	0.3078	-	**Cramer's V**	-	0.3001	-
**Table Probability**	-	-	<.0001	**Table Probability**	-	-	.0004
**Pr <= P**	-	-	.0538	**Pr <= P**	-	-	.0126
**Control (no vernalisation)**							
**Distance to centre of UHI**							
**Log likelihood**	**AIC**	**AICC**	**BIC**				
-47.5317	99.0635	99.1847	104.3134				
**Parameter**	**Degrees of Freedom**	**Estimate**	**Standard Error**	**Likelihood Ratio 95% Confidence Limits**		**Wald Chi-Square**	**Pr > ChiSq**
**Intercept**	1	-1.5493	0.5301	-2.6776	-0.5744	8.54	.0035
**Distance**	1	0.0000	0.0001	-0.0001	0.0001	0.00	.9847
**Scale**	0	1.0000	0.0000	1.0000	1.0000		
**Subhabitat**				**District**			
	**Degrees of Freedom**	**Value**	**Probability**		**Degrees of Freedom**	**Value**	**Probability**
**Chi-Square**	4	7.1700	.1272	**Chi-Square**	2	4.9582	.0838
**Likelihood Ratio Chi-Square**	4	6.6873	.1534	**Likelihood Ratio Chi-Square**	2	6.2649	.0436
**Mantel-Haenszel Chi-Square**	1	2.1525	.1423	**Mantel-Haenszel Chi-Square**	1	0.0342	.8532
**Phi coefficient**	-	0.2651	-	**Phi coefficient**	-	0.2205	-
**Contingency coefficient**	-	0.2563	-	**Contingency coefficient**	-	0.2153	-
**Cramer's V**	-	0.2651	-	**Cramer's V**	-	0.2205	-
**Table Probability**	-	-	.0003	**Table Probability**	-	-	.0039
**Pr <= P**	-	-	.1289	**Pr <= P**	-	-	.0652

Table S5.1: Results of the statistics analyses for the heat treatment experiment. All analyses were performed using generalised linear models (Methods section 4 for details) with distance to the start of the urban-rural transect as a continuous variable, and subhabitat and district (Methods section 1 for definitions) as categorical variables.

PROC GLM, dist_start_trans, biomass_tot

The GLM Procedure

Class Level Information		
Class	Levels	Values
Accession	86	10_1 10_3 11_1 11_2 11_3 12_1 12_2 12_3 13_1 13_2 13_3 14_1 14_2 16_2 16_3 17_1 17_2 17_3 18_1 18_3 19_1 19_3 1_1 1_2 1_3 20_2 20_3 21_1 21_2 21_3 22_1 22_2 23_2 23_3 24_1 24_3 25_1 25_2 25_3 26_1 26_3 27_1 27_3 28_1 29_1 29_3 2_1 2_2 2_3 30_1 30_2 30_3 31_1 31_3 32_1 32_2 32_3 33_2 35_2 35_3 36_1 36_2 36_3 37_2 38_2 3_1 3_2 3_3 40_1 40_2 40_3 4_3 5_1 5_2 5_3 6_1 6_2 6_3 7_1 7_2 7_3 8_1 8_2 8_3 9_1 9_3
temp	4	20C 26C 32C 38C
rep	3	1 2 3
city	3	I II III
microh	5	I II III IV V

Number of Observations Read	954
Number of Observations Used	942

PROC GLM, dist_start_trans, biomass_tot

The GLM Procedure

Dependent Variable: biomass_tot

Source	DF	Sum of Squares	Mean Square	F Value	Pr > F
Model	10	935882.894	93588.289	382.39	<.0001
Error	931	227856.178	244.743		
Corrected Total	941	1163739.072			

R-Square	Coeff Var	Root MSE	biomass_tot Mean
0.804203	24.75851	15.64428	63.18747

Source	DF	Type I SS	Mean Square	F Value	Pr > F
rep	2	2696.0491	1348.0245	5.51	0.0042
temp	3	790967.5841	263655.8614	1077.27	<.0001
init_leaflength	1	136814.8100	136814.8100	559.01	<.0001
dist_start_trans	1	2324.2111	2324.2111	9.50	0.0021
dist_start_tran*temp	3	3080.2395	1026.7465	4.20	0.0058

Source	DF	Type III SS	Mean Square	F Value	Pr > F
rep	2	47246.5879	23623.2940	96.52	<.0001
temp	3	243857.7938	81285.9313	332.13	<.0001
init_leaflength	1	137360.2742	137360.2742	561.24	<.0001
dist_start_trans	1	2381.5456	2381.5456	9.73	0.0019
dist_start_tran*temp	3	3080.2395	1026.7465	4.20	0.0058

PROC GLM, city effect, biomass_tot

The GLM Procedure

Class Level Information		
Class	Levels	Values
Accession	86	10_1 10_3 11_1 11_2 11_3 12_1 12_2 12_3 13_1 13_2 13_3 14_1 14_2 16_2 16_3 17_1 17_2 17_3 18_1 18_3 19_1 19_3 1_1 1_2 1_3 20_2 20_3 21_1 21_2 21_3 22_1 22_2 23_2 23_3 24_1 24_3 25_1 25_2 25_3 26_1 26_3 27_1 27_3 28_1 29_1 29_3 2_1 2_2 2_3 30_1 30_2 30_3 31_1 31_3 32_1 32_2 32_3 33_2 35_2 35_3 36_1 36_2 36_3 37_2 38_2 3_1 3_2 3_3 40_1 40_2 40_3 4_3 5_1 5_2 5_3 6_1 6_2 6_3 7_1 7_2 7_3 8_1 8_2 8_3 9_1 9_3
temp	4	20C 26C 32C 38C

Class Level Information		
Class	Levels	Values
rep	3	1 2 3
city	3	I II III
microh	5	I II III IV V

Number of Observations Read	954
Number of Observations Used	942

PROC GLM, city effect, biomass_tot

The GLM Procedure

Dependent Variable: biomass_tot

Source	DF	Sum of Squares	Mean Square	F Value	Pr > F
Model	14	936433.901	66888.136	272.78	<.0001
Error	927	227305.172	245.205		
Corrected Total	941	1163739.072			

R-Square	Coeff Var	Root MSE	biomass_tot Mean
0.804677	24.78185	15.65903	63.18747

Source	DF	Type I SS	Mean Square	F Value	Pr > F
rep	2	2696.0491	1348.0245	5.50	0.0042
temp	3	790967.5841	263655.8614	1075.25	<.0001
init_leaflength	1	136814.8100	136814.8100	557.96	<.0001
city	2	2627.3743	1313.6872	5.36	0.0049
temp*city	6	3328.0831	554.6805	2.26	0.0357

Source	DF	Type III SS	Mean Square	F Value	Pr > F
rep	2	47218.6757	23609.3378	96.28	<.0001
temp	3	766527.5844	255509.1948	1042.02	<.0001
init_leaflength	1	136294.3791	136294.3791	555.84	<.0001
city	2	2736.3177	1368.1588	5.58	0.0039
temp*city	6	3328.0831	554.6805	2.26	0.0357

PROC GLM, city effect, biomass_tot

The GLM Procedure
Least Squares Means
Adjustment for Multiple Comparisons: Tukey-Kramer

city	biomass_tot LSMEAN	LSMEAN Number
I	66.1962666	1
II	63.8709636	2
III	62.0407175	3

Least Squares Means for effect city
Pr > |t| for H0: LSMean(i)=LSMean(j)

Dependent Variable: biomass_tot

i/j	1	2	3
1		0.2147	0.0026
2	0.2147		0.2981

Least Squares Means for effect city
Pr > |t| for H0: LSMean(i)=LSMean(j)

Dependent Variable: biomass_tot

i/j	1	2	3
3	0.0026	0.2981	

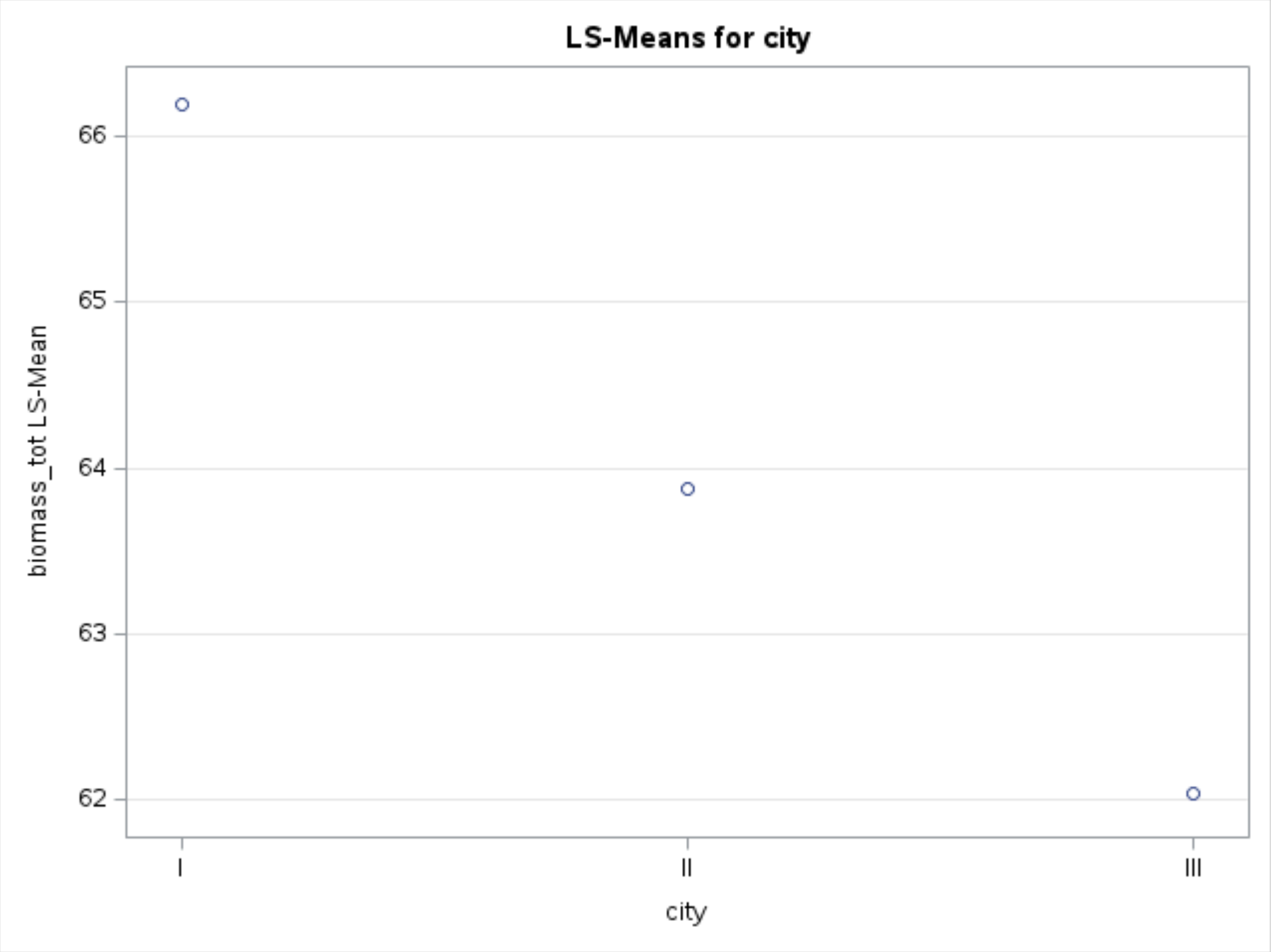

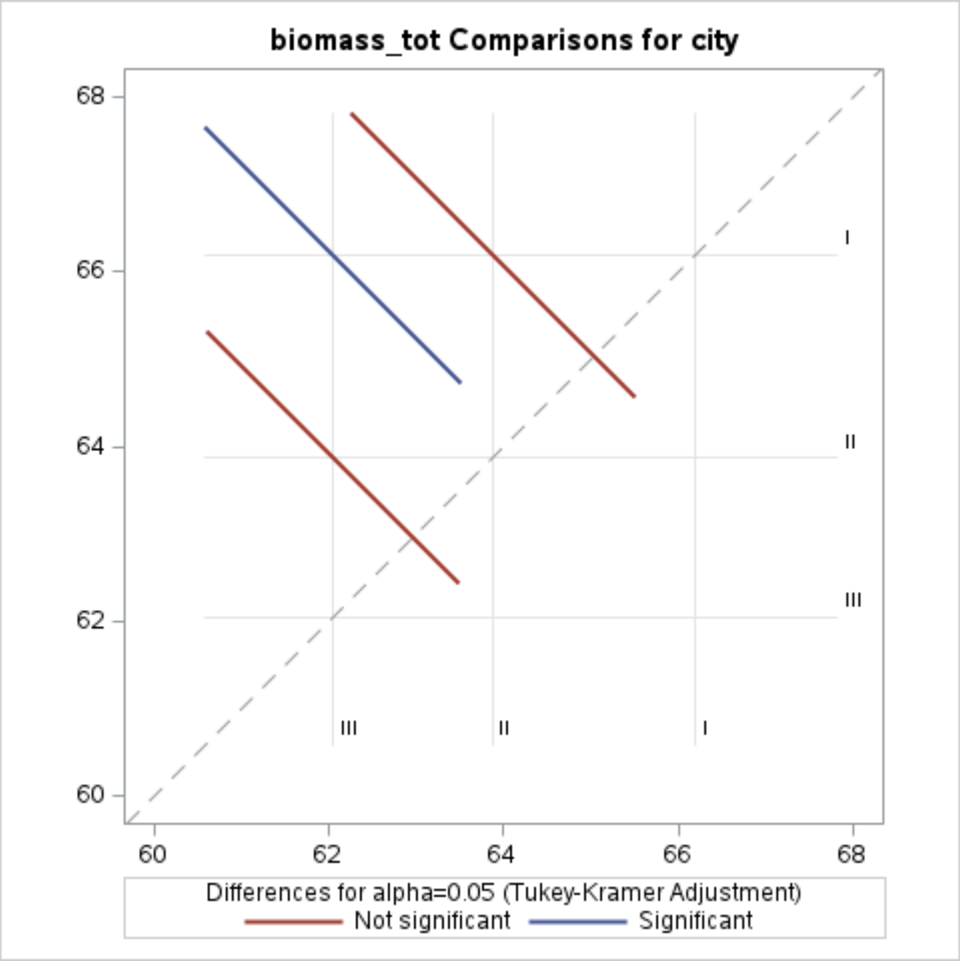

PROC GLM, microh effect, biomass_tot

The GLM Procedure

Class Level Information		
**Class**	**Levels**	**Values**
**Accession**	86	10_1 10_3 11_1 11_2 11_3 12_1 12_2 12_3 13_1 13_2 13_3 14_1 14_2 16_2 16_3 17_1 17_2 17_3 18_1 18_3 19_1 19_3 1_1 1_2 1_3 20_2 20_3 21_1 21_2 21_3 22_1 22_2 23_2 23_3 24_1 24_3 25_1 25_2 25_3 26_1 26_3 27_1 27_3 28_1 29_1 29_3 2_1 2_2 2_3 30_1 30_2 30_3 31_1 31_3 32_1 32_2 32_3 33_2 35_2 35_3 36_1 36_2 36_3 37_2 38_2 3_1 3_2 3_3 40_1 40_2 40_3 4_3 5_1 5_2 5_3 6_1 6_2 6_3 7_1 7_2 7_3 8_1 8_2 8_3 9_1 9_3
**temp**	4	20C 26C 32C 38C
**rep**	3	1 2 3
**city**	3	I II III
**microh**	5	I II III IV V

**Number of Observations Read**	954
**Number of Observations Used**	942

PROC GLM, microh effect, biomass_tot

The GLM Procedure

Dependent Variable: biomass_tot

Source	DF	Sum of Squares	Mean Square	F Value	Pr > F
**Model**	22	938519.742	42659.988	174.07	<.0001
**Error**	919	225219.330	245.070		
**Corrected Total**	941	1163739.072			

R-Square	Coeff Var	Root MSE	biomass_tot Mean
0.806469	24.77502	15.65471	63.18747

Source	DF	Type I SS	Mean Square	F Value	Pr > F
rep	2	2696.0491	1348.0245	5.50	0.0042
temp	3	790967.5841	263655.8614	1075.84	<.0001
init_leaflength	1	136814.8100	136814.8100	558.27	<.0001
microh	4	2524.5258	631.1314	2.58	0.0363
temp*microh	12	5516.7727	459.7311	1.88	0.0337

Source	DF	Type III SS	Mean Square	F Value	Pr > F
rep	2	45821.3115	22910.6557	93.49	<.0001
temp	3	609409.1407	203136.3802	828.89	<.0001
init_leaflength	1	132707.3015	132707.3015	541.51	<.0001
microh	4	2630.4981	657.6245	2.68	0.0304
temp*microh	12	5516.7727	459.7311	1.88	0.0337

Table S5.2: Results of the linear model analyses for the heat treatment experiment. Linear models were fit separately for each temperature treatment with distance to the start of the urban-rural transect as a continuous variable.

PROC GLM, dist_start_trans, biomass_tot

The GLM Procedure

Dependent Variable: biomass_tot

temp=20C

Source	DF	Sum of Squares	Mean Square	F Value	Pr > F
Model	4	38270.57344	9567.64336	67.60	<.0001
Error	239	33824.94426	141.52696		
Corrected Total	243	72095.51770			

R-Square	Coeff Var	Root MSE	biomass_tot Mean
0.530832	20.14063	11.89651	59.06721

Source	DF	Type I SS	Mean Square	F Value	Pr > F
rep	2	15867.71900	7933.85950	56.06	<.0001
init_leaflength	1	22382.98680	22382.98680	158.15	<.0001
dist_start_trans	1	19.86764	19.86764	0.14	0.7082

Source	DF	Type III SS	Mean Square	F Value	Pr > F
rep	2	7760.50400	3880.25200	27.42	<.0001
init_leaflength	1	22028.51100	22028.51100	155.65	<.0001
dist_start_trans	1	19.86764	19.86764	0.14	0.7082

Parameter	Estimate		Standard Error	t Value	Pr > |t|
Intercept	-0.27847660	B	4.70386035	-0.06	0.9528
rep 1	9.12794003	B	2.16009672	4.23	<.0001
rep 2	13.63015536	B	1.87037687	7.29	<.0001
rep 3	0.00000000	B	.	.	.
init_leaflength	3.26137869		0.26141364	12.48	<.0001
dist_start_trans	0.00007883		0.00021038	0.37	0.7082

Note: The X'X matrix has been found to be singular, and a generalized inverse was used to solve the normal equations. Terms whose estimates are followed by the letter 'B' are not uniquely estimable.

PROC GLM, dist_start_trans, biomass_tot

The GLM Procedure

temp=26C

Class Level Information

Class	Levels	Values
Accession	86	10_1 10_3 11_1 11_2 11_3 12_1 12_2 12_3 13_1 13_2 13_3 14_1 14_2 16_2 16_3 17_1 17_2 17_3 18_1 18_3 19_1 19_3 1_1 1_2 1_3 20_2 20_3 21_1 21_2 21_3 22_1 22_2 23_2 23_3 24_1 24_3 25_1 25_2 25_3 26_1 26_3 27_1 27_3 28_1 29_1 29_3 2_1 2_2 2_3 30_1 30_2 30_3 31_1 31_3 32_1 32_2 32_3 33_2 35_2 35_3 36_1 36_2 36_3 37_2 38_2 3_1 3_2 3_3 40_1 40_2 40_3 4_3 5_1 5_2 5_3 6_1 6_2 6_3 7_1 7_2 7_3 8_1 8_2 8_3 9_1 9_3
temp	1	26C
rep	3	1 2 3
city	3	I II III
microh	5	I II III IV V

Number of Observations Read	233
Number of Observations Used	226

PROC GLM, dist_start_trans, biomass_tot

The GLM Procedure

Dependent Variable: biomass_tot

temp=26C

Source	DF	Sum of Squares	Mean Square	F Value	Pr > F
Model	4	123000.5604	30750.1401	98.37	<.0001
Error	221	69086.6589	312.6093		
Corrected Total	225	192087.2193			

R-Square	Coeff Var	Root MSE	biomass_tot Mean
0.640337	18.76903	17.68076	94.20177

Source	DF	Type I SS	Mean Square	F Value	Pr > F
rep	2	35074.59162	17537.29581	56.10	<.0001

Source	DF	Type I SS	Mean Square	F Value	Pr > F
init_leaflength	1	84057.51994	84057.51994	268.89	<.0001
dist_start_trans	1	3868.44886	3868.44886	12.37	0.0005

Source	DF	Type III SS	Mean Square	F Value	Pr > F
rep	2	36418.68051	18209.34026	58.25	<.0001
init_leaflength	1	85682.98466	85682.98466	274.09	<.0001
dist_start_trans	1	3868.44886	3868.44886	12.37	0.0005

Parameter	Estimate		Standard Error	t Value	Pr > |t|
Intercept	3.24054963	B	6.37159849	0.51	0.6115
rep 1	29.81617179	B	2.94903586	10.11	<.0001
rep 2	6.56379290	B	3.13227171	2.10	0.0373
rep 3	0.00000000	B	.	.	.
init_leaflength	5.92131048		0.35766093	16.56	<.0001
dist_start_trans	-0.00112402		0.00031953	-3.52	0.0005

Note: The X'X matrix has been found to be singular, and a generalized inverse was used to solve the normal equations. Terms whose estimates are followed by the letter 'B' are not uniquely estimable.

PROC GLM, dist_start_trans, biomass_tot

The GLM Procedure

temp=32C

Class Level Information

Class	Levels	Values
Accession	86	10_1 10_3 11_1 11_2 11_3 12_1 12_2 12_3 13_1 13_2 13_3 14_1 14_2 16_2 16_3 17_1 17_2 17_3 18_1 18_3 19_1 19_3 1_1 1_2 1_3 20_2 20_3 21_1 21_2 21_3 22_1 22_2 23_2 23_3 24_1 24_3 25_1 25_2 25_3 26_1 26_3 27_1 27_3 28_1 29_1 29_3 2_1 2_2 2_3 30_1 30_2 30_3 31_1 31_3 32_1 32_2 32_3 33_2 35_2 35_3 36_1 36_2 36_3 37_2 38_2 3_1 3_2 3_3 40_1 40_2 40_3 4_3 5_1 5_2 5_3 6_1 6_2 6_3 7_1 7_2 7_3 8_1 8_2 8_3 9_1 9_3
temp	1	32C
rep	3	1 2 3
city	3	I II III
microh	5	I II III IV V

Number of Observations Read	238
Number of Observations Used	237

PROC GLM, dist_start_trans, biomass_tot

The GLM Procedure

Dependent Variable: biomass_tot

temp=32C

Source	DF	Sum of Squares	Mean Square	F Value	Pr > F
Model	4	27582.5391	6895.6348	22.05	<.0001
Error	232	72541.8533	312.6804		
Corrected Total	236	100124.3924			

R-Square	Coeff Var	Root MSE	biomass_tot Mean
0.275483	21.39460	17.68277	82.65063

Source	DF	Type I SS	Mean Square	F Value	Pr > F
rep	2	5916.66920	2958.33460	9.46	0.0001

Source	DF	Type I SS	Mean Square	F Value	Pr > F
init_leaflength	1	20633.26849	20633.26849	65.99	<.0001
dist_start_trans	1	1032.60145	1032.60145	3.30	0.0705

Source	DF	Type III SS	Mean Square	F Value	Pr > F
rep	2	15030.90700	7515.45350	24.04	<.0001
init_leaflength	1	20936.30568	20936.30568	66.96	<.0001
dist_start_trans	1	1032.60145	1032.60145	3.30	0.0705

Parameter	Estimate		Standard Error	t Value	Pr > |t|
Intercept	32.33473239	B	6.32706831	5.11	<.0001
rep 1	22.08604355	B	3.30749637	6.68	<.0001
rep 2	12.92307981	B	2.79043534	4.63	<.0001
rep 3	0.00000000	B	.	.	.
init_leaflength	2.68486843		0.32811291	8.18	<.0001
dist_start_trans	-0.00056976		0.00031353	-1.82	0.0705

Note: The X'X matrix has been found to be singular, and a generalized inverse was used to solve the normal equations. Terms whose estimates are followed by the letter 'B' are not uniquely estimable.

PROC GLM, dist_start_trans, biomass_tot

The GLM Procedure

temp=38C

Class Level Information		
Class	Levels	Values
Accession	85	10_1 10_3 11_1 11_2 11_3 12_1 12_2 12_3 13_1 13_2 13_3 14_1 14_2 16_2 16_3 17_1 17_2 17_3 18_1 18_3 19_1 19_3 1_1 1_2 1_3 20_2 20_3 21_1 21_2 21_3 22_1 22_2 23_2 23_3 24_1 24_3 25_1 25_2 25_3 26_1 26_3 27_1 27_3 28_1 29_1 29_3 2_1 2_2 2_3 30_1 30_2 31_1 31_3 32_1 32_2 32_3 33_2 35_2 35_3 36_1 36_2 36_3 37_2 38_2 3_1 3_2 3_3 40_1 40_2 40_3 4_3 5_1 5_2 5_3 6_1 6_2 6_3 7_1 7_2 7_3 8_1 8_2 8_3 9_1 9_3
temp	1	38C
rep	3	1 2 3
city	3	I II III
microh	5	I II III IV V

Number of Observations Read	239
Number of Observations Used	235

PROC GLM, dist_start_trans, biomass_tot

The GLM Procedure

Dependent Variable: biomass_tot

temp=38C

Source	DF	Sum of Squares	Mean Square	F Value	Pr > F
Model	4	3448.184545	862.046136	39.30	<.0001
Error	230	5044.610944	21.933091		
Corrected Total	234	8492.795489			

R-Square	Coeff Var	Root MSE	biomass_tot Mean
0.406013	26.00346	4.683278	18.01021

Source	DF	Type I SS	Mean Square	F Value	Pr > F
rep	2	1237.708844	618.854422	28.22	<.0001
init_leaflength	1	2204.150615	2204.150615	100.49	<.0001

Source	DF	Type I SS	Mean Square	F Value	Pr > F
dist_start_trans	1	6.325086	6.325086	0.29	0.5918

Source	DF	Type III SS	Mean Square	F Value	Pr > F
rep	2	417.432560	208.716280	9.52	0.0001
init_leaflength	1	2206.522012	2206.522012	100.60	<.0001
dist_start_trans	1	6.325086	6.325086	0.29	0.5918

Parameter	Estimate		Standard Error	t Value	Pr > |t|
Intercept	0.500776372	B	2.15737178	0.23	0.8166
rep 1	3.089015221	B	0.93764510	3.29	0.0011
rep 2	0.081395580	B	0.94794011	0.09	0.9316
rep 3	0.000000000	B	.	.	.
init_leaflength	1.118152714		0.11147999	10.03	<.0001
dist_start_trans	-0.000045151		0.00008408	-0.54	0.5918

Note: The X'X matrix has been found to be singular, and a generalized inverse was used to solve the normal equations. Terms whose estimates are followed by the letter 'B' are not uniquely estimable.

PROC GLM, dist_start_trans, biomass_tot

Obs	Dependent	Parameter	Estimate	Biased	StdErr	tValue	Probt
1	biomass_tot	Intercept	-45.42914796	1	3.36922022	-13.48	<.0001
2	biomass_tot	rep 1	19.41347042	1	1.39753964	13.89	<.0001
3	biomass_tot	rep 2	9.80966308	1	1.28954452	7.61	<.0001
4	biomass_tot	rep 3	0.00000000	1	.	.	.
5	biomass_tot	temp 20C	37.56327038	1	2.81490631	13.34	<.0001
6	biomass_tot	temp 26C	84.88506458	1	2.87245379	29.55	<.0001
7	biomass_tot	temp 32C	65.37857410	1	2.81900763	23.19	<.0001
8	biomass_tot	temp 38C	0.00000000	1	.	.	.
9	biomass_tot	init_leaflength	3.61133597	0	0.15243787	23.69	<.0001
10	biomass_tot	dist_start_trans	-0.00001904	1	0.00028020	-0.07	0.9458
11	biomass_tot	dist_start_tran*temp 20C	0.00008100	1	0.00039305	0.21	0.8368
12	biomass_tot	dist_start_tran*temp 26C	-0.00114314	1	0.00039705	-2.88	0.0041
13	biomass_tot	dist_start_tran*temp 32C	-0.00060162	1	0.00039416	-1.53	0.1273
14	biomass_tot	dist_start_tran*temp 38C	0.00000000	1	.	.	.
